# 3D structure and in situ arrangements of CatSper channel in the sperm flagellum

**DOI:** 10.1038/s41467-022-31050-8

**Published:** 2022-06-17

**Authors:** Yanhe Zhao, Huafeng Wang, Caroline Wiesehoefer, Naman B. Shah, Evan Reetz, Jae Yeon Hwang, Xiaofang Huang, Tse-en Wang, Polina V. Lishko, Karen M. Davies, Gunther Wennemuth, Daniela Nicastro, Jean-Ju Chung

**Affiliations:** 1 Department of Cell Biology, University of Texas Southwestern Medical Center, Dallas, TX 75390 USA; 2 Department of Cellular and Molecular Physiology, Yale School of Medicine, New Haven, CT 06510 USA; 3 Department of Anatomy, University of Duisburg-Essen, Medical Faculty, 45147 Essen, Germany; 4 Department of Molecular and Cell Biology, University of California, Berkeley, CA 94720 USA; 5 Molecular Biophysics and Integrative Bioimaging division, Bioscience Area, Lawrence Berkeley National Laboratory, Berkeley, CA 94720 USA; 6 The Center for Reproductive Longevity and Equality, Buck Institute for Research on Aging, Novato, CA 94945 USA; 7 Department of Obstetrics, Gynecology, and Reproductive Sciences, Yale School of Medicine, New Haven, CT 06510 USA

**Keywords:** Cryoelectron tomography, Calcium signalling, Reproductive biology, Calcium channels

## Abstract

The sperm calcium channel CatSper plays a central role in successful fertilization as a primary Ca^2+^ gateway. Here, we applied cryo-electron tomography to visualize the higher-order organization of the native CatSper complex in intact mammalian sperm. The repeating CatSper units form long zigzag-rows along mouse and human sperm flagella. Above each tetrameric channel pore, most of the extracellular domains form a canopy that interconnects to a zigzag-shaped roof. Murine CatSper contains an additional wing-structure connected to the tetrameric channel. The intracellular domains link two neighboring channels to a diagonal array, suggesting a dimer formation. Fitting of an atomic model of isolated monomeric CatSper to the in situ map reveals supramolecular interactions and assembly of the CatSper complex. Loss of EFCAB9-CATSPERζ alters the architecture and interactions of the channels, resulting in fragmentation and misalignment of the zigzag-rows and disruption of flagellar movement in *Efcab9*^−/−^ sperm. This work offers unique insights into the structural basis for understanding CatSper regulation of sperm motility.

## Abstract (web summary)

Sperm motility and male fertility requires function of the CatSper calcium channels. Here, using cryo-electron tomography, authors visualize the native in-cell 3D structure and higher-order organization of the CatSper as long zigzag rows along the sperm tail.

## Introduction

Freshly ejaculated mammalian sperm must undergo a physiological process called capacitation to be capable of fertilizing the egg^1,2^. The crucial change that occurs during capacitation represents a motility change, i.e., the sperm flagellum beats vigorously and asymmetrically, producing a whip-like motion. This motility pattern—known as hyperactivated motility—enables the sperm to reach the egg by overcoming the viscous microenvironment of the female reproductive tract. In addition, hyperactivation allows sperm to push through a sticky egg coat, and eventually fertilize the egg^3^. Hyperactivation is triggered by the elevation of the intraflagellar calcium that requires the sperm-specific and Ca^2+^-selective CatSper ion channel^4,5^. CatSper loss-of-function abrogates hyperactivation of the sperm flagellum and renders males infertile in both mice and humans^6^.

Previous studies have found that CatSper is one of the most complex ion channels known, comprised of at least ten proteins: four subunits that form a heterotetrameric channel (CATSPER1-4)^5,7^, as well as six accessory, non-pore forming subunits, including four transmembrane (TM) proteins (CATSPERβ, γ, δ, and ε)^8–11^ with large extracellular domains (ECD) and two small cytoplasmic proteins, EFCAB9 and CATSPERζ^8,12^, that form a calmodulin (CaM)-IQ domain subcomplex^13^ (Supplementary Table 1). Up to now, deletions or mutations of all the reported pore-forming or other TM subunits results in the loss of the entire CatSper channel complex in the sperm flagella in mice^6^. Super-resolution light microscopy showed that the CatSper channel complex is restricted to four linear compartments within the flagellar membrane in both mouse^8,12,14,15^ and human sperm^8,16^, generating a unique longitudinal signaling nanodomain in each flagellar quadrant. Genetic evidence^8,12,14^ and molecular imaging of sperm distributed along the female tract^17^ suggested that this unique arrangement is essential for Ca^2+^ signaling and sperm hyperactivation for successful fertilization, highlighting physiological relevance of the spatial organization. Disrupting the integrity of the linear nanodomains alters the flagellar waveform and prevents sperm from efficiently migrating in vivo^8,14,17^. Specifically, the absence of the cytoplasmic EFCAB9-CATSPERζ complex in *Efcab9*^−/−^ and/or *Catsperz*^−/−^ mutant sperm alters the continuity of each CatSper nanodomain^8,12^, suggesting a regularly repeating, quaternary structure of the CatSper complex within the nanodomains.

Despite many important discoveries mentioned above, the fundamental structure of the native channel complex and its molecular architectural arrangement are just beginning to emerge^18,19^. Here, we address these questions by visualizing the in-cell higher-order organization and domain structures of the CatSper channel complex in intact mouse and human sperm flagella using cryo-electron tomography (cryo-ET). We observed dimers of two staggered channel units that form long zigzag rows along the sperm flagella. Fitting the atomic model of isolated monomeric CatSper complex^18^ into our cryo-ET in situ density map further reveals unprecedented structural insights into the interfaces within and between the CatSper channel dimeric complexes.

## Results and discussion

### In-cell organization of CatSper complexes

**Fig. 1:**
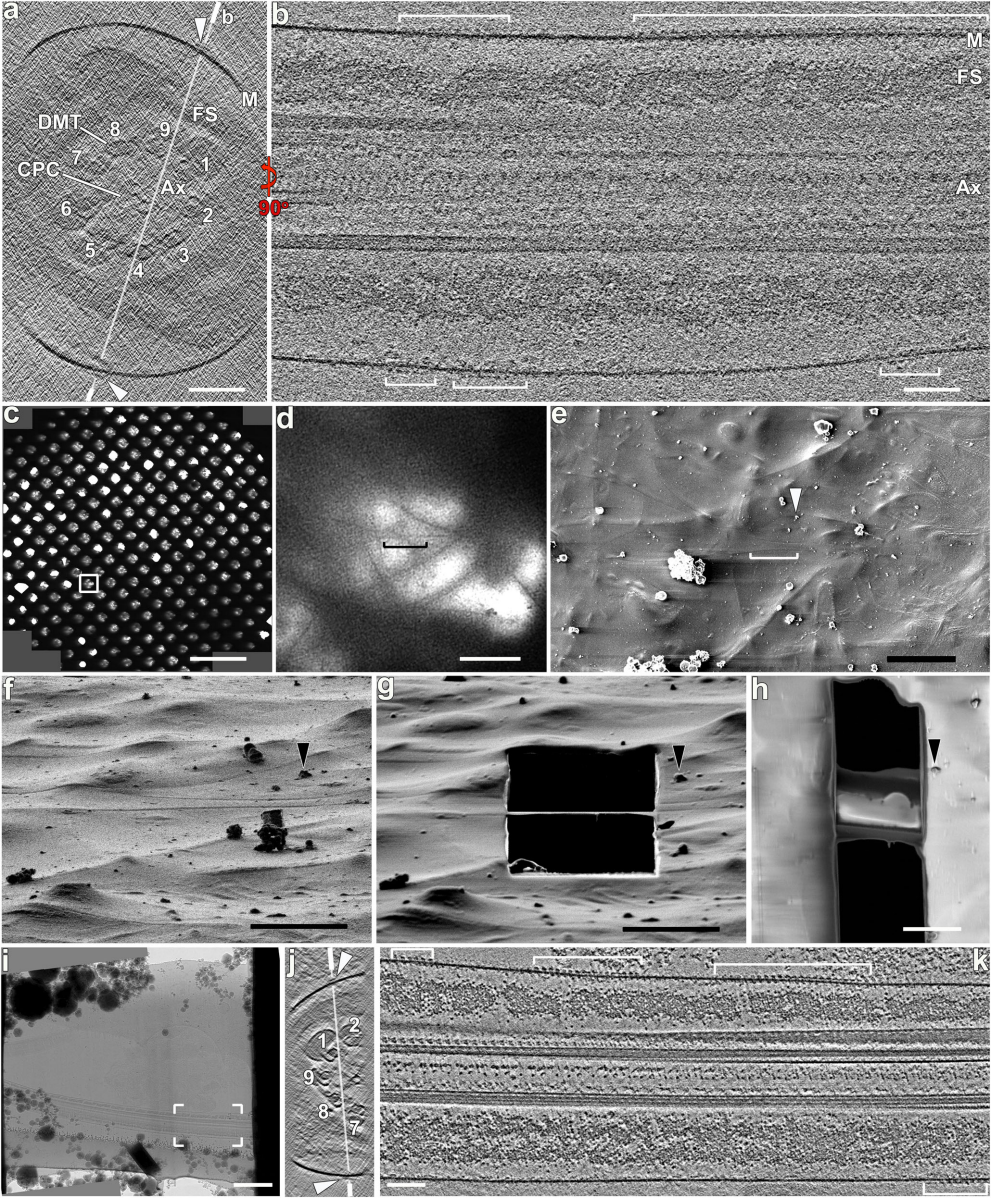
Cryo-ET of whole-cell and cryo-FIB milled mouse sperm flagella reveals rows of CatSper complex particles. **a**–**b** Cross-sectional (**a**) and longitudinal (**b**) tomographic slices of a representative principal piece region from a whole-cell (i.e., not cryo-FIB milled) wild type mouse sperm flagellum (in non-capacitated state) show CatSper complexes (arrowheads, brackets). Labeled line in (**a**) indicates the position of the section shown in (**b**). **c**–**h** The cryo-FIB milling workflow consists of: selection of a suitable sample area using an overview (**c**) and zoom-in (**d**) cryo-TEM image of an EM grid with plunge-frozen mouse sperm; scanning-EM image recorded in the Dual-Beam/FIB instrument (**e**) showing the same grid area as in (**d**); SEM images then show the targeted sperm flagellum before (**f**) and after (**g**, **h**) cryo-FIB milling from top (**e**, **h**) and side (**f**, **g**) views. For reference, the same particle (black arrowheads) is indicated in all SEM images. **i**–**k** A cryo-TEM image (**i**) of the same cryo-FIB lamella shown in (**h**) allows selecting an area with a mouse sperm flagellum (boxed area) for tilt series recording. Cross-sectional (**j**) and longitudinal (**k**) tomographic slices show better contrast and resolution for the cryo-FIB milled flagellum and CatSper complexes (arrowheads) than for whole-cell samples (compare **j**, **k** to **a**, **b**). Labeled line in (**j**) indicates the position of the section shown in (**k**). Other labels: Ax axoneme, CPC central pair complex, DMT & 1-9, doublet microtubules, FS fibrous sheath, M membrane. Axonemal structures are shown in cross-sectional views from proximal to distal orientation, and in longitudinal views with proximal side of the flagellum on the left in all figures unless otherwise noted. Scale bars: 100 nm in (**a**, **b**, **j**, **k**); 400 μm in (**c**); 10 μm in (**d**, **e**); 4 μm in (**f**–**h**); 1 μm in (**i**).

**Fig. 2:**
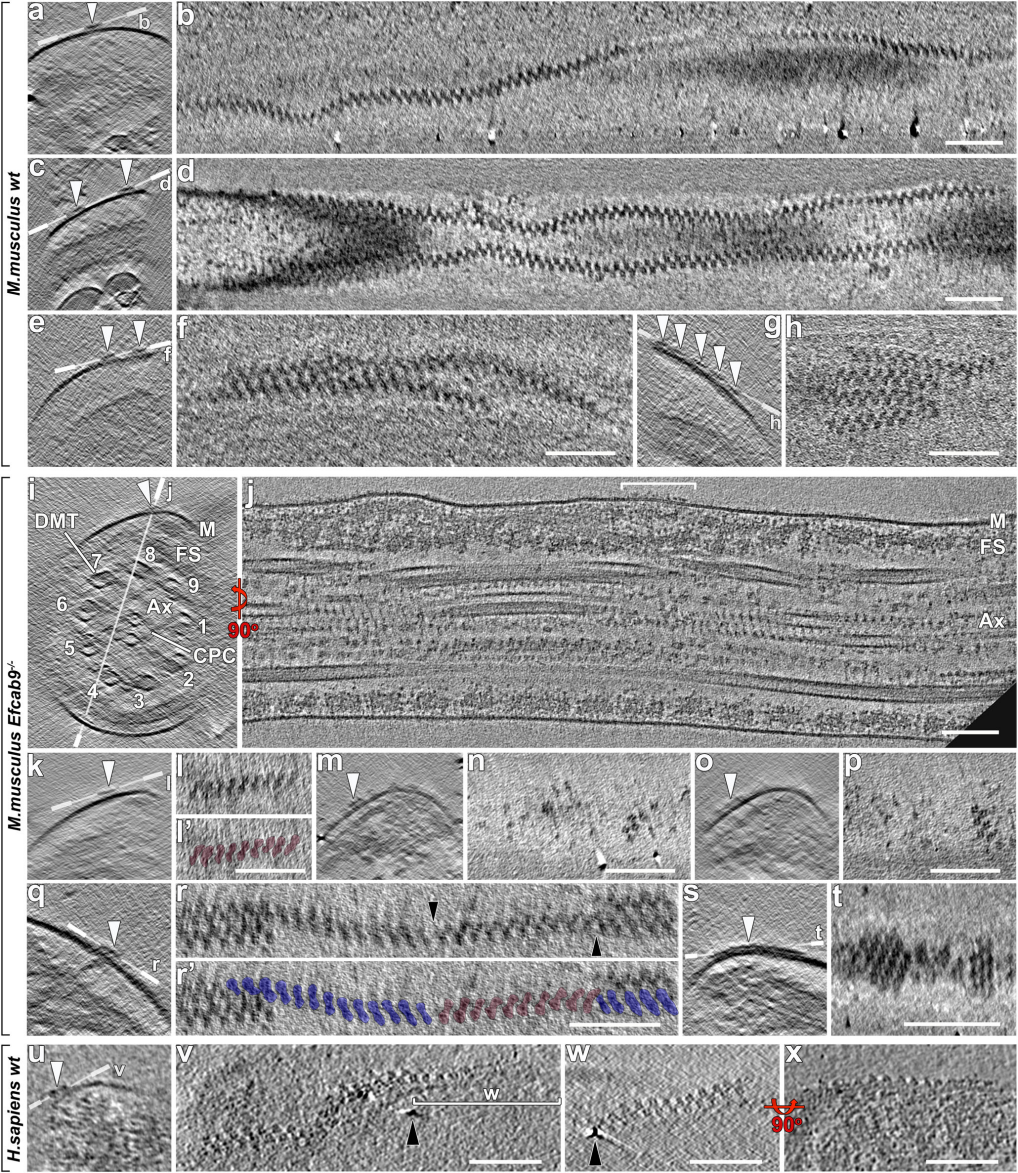
In-cell structure of the native CatSper complexes in intact mammalian sperm flagella. **a**–**h** Representative tomographic slices of the repeating CatSper channel complexes arranged in zigzag-rows along the longitudinal axis of wild type mouse sperm flagella shown as cross-sectional (**a**, **c**, **e**, **g**) and top views (**b**, **d**, **f**, **h**). The number of zigzag-rows (arrowheads) varied from a single row (**a**, **b**), two rows (**c**, **d**), merging rows (**e**, **f**), to up to five rows (**g**, **h**). **i**–**j** A representative principal piece region of a whole-cell (i.e., not cryo-FIB milled) *Efcab9*^−/−^ mouse sperm flagellum (in non-capacitated state) viewed in cross-sectional (**i**) and longitudinal (**j**) tomographic slices. Labeled line in (**i**) indicates the position of the section shown in (**j**). Other labels: Ax axoneme, CPC central pair complex, DMT & 1-9 doublet microtubules, FS fibrous sheath, M membrane. **k**–**t** Representative tomographic slices from *Efcab9*^−/−^ sperm showing fragmented and short CatSper complex clusters with altered orientation relative to the flagellar axis (cross section views: **k**, **m**, **o**, **q**, **s**; top views: **l**. **l**’, **n**, **p**, **r**, **r**’, **t**). Two distinct arrangements are pseudo-colored (backslash (\) blue and forward slash (/) pink) in (**l**’ and **r**’). Black arrowheads in (**r**) indicate the interruptions of the row. **u**–**x** Zigzag-arrangement of CatSper in a human sperm flagellum shown in cross-sectional (**u**), top (**v**, **w**) and side view (**x**). Black arrowheads in (**v** and **w**) indicate the same position between the two slices show at slightly different angles. Scale bars, 100 nm.

Ten components have been validated to comprise the CatSper channel complex in the linear nanodomains along sperm flagella^6^. Based on the complexity of the known CatSper components, and the reported 1:1 stoichiometry of TM CatSper subunits in sea urchin sperm (i.e., CATSPER1-4, β, γ, and ε)^20^, we hypothesized that a single mouse or human CatSper complex forms a nearly half-megadalton extracellular domain (ECD) (Supplementary Table 1), which could be visualized by cellular cryo-ET. Therefore, we performed cryo-ET on intact murine and human sperm flagella to characterize the native CatSper complex in situ, which avoids potential purification artifacts and could inform about their higher-order molecular organization. Viewing the 3D reconstructed sperm and flagellar membranes in cross-section, we indeed observed protruding particles of ~25 nm in width positioned to either side of the longitudinal column of the fibrous sheath in the principal piece (Fig. 1a), i.e., close to doublet numbers 2, 4, 7 or 9, which is consistent with the localization for CatSper nanodomains as previously seen by immuno-electron microscopy (EM)^14^. Out of the four quadrants, only up to two could be visualized in the cryo-tomograms due to the missing wedge effect that results from a limited tilt-angle range in single-axis ET. Longitudinal tomographic slices of the wild type sperm flagella revealed long continuous rows of densely packed particles with an apparent periodicity of 17.6 nm (Fig. 1b). The signal-to-noise ratio and thus resolution of these reconstructed whole murine flagella was relatively low due to the ~900 nm sample thickness of the proximal principal piece of mouse sperm flagellum which contains a high density of CatSper channels^14^. Therefore, we also used cryo-focused ion beam (cryo-FIB) milling to generate ~200 nm thick slices (called “lamella”) of murine sperm flagella (Fig. 1c–i) that resulted in higher-resolution tomographic reconstructions (Fig. 1j, k, Supplementary Table 2). Slicing the rows of the protruding particles in longitudinal orientation parallel to the flagellar membrane (top view) revealed continuous rows with repeating units in a zigzag arrangement of ~25 nm in width (Fig. 2a–h), demonstrating the protein complexes are repeated within the rows. We found that the number of zigzag rows per nanodomain varies from a single row (Fig. 2a, b), two rows that can be up to 100 nm apart (Fig. 2c, d, Supplementary Movie 1) or fuse to one row (Fig. 2e, f), and up to as many as five parallel rows (Fig. 2g, h). From the 18 observed nanodomains in 11 cryo-tomograms of wild type flagella (Supplementary Table 2), we found the following number of zigzag rows: 1 row (15 times), 2 rows (8 times), 4 rows (1 time), and 5 rows (1 time).

To ensure that the observed zigzag structure is indeed formed by CatSper complexes, we chose a wild type-mutant comparison approach. Due to all-or-none assembly of CatSper TM subunits in mice, knockout of any one of these TM subunits leads to a loss of the entire CatSper complex, thus lacking the nanodomains^6^ (also see below). Considering the anisotropic resolution inherent to single-axis cryo-ET and the relatively small fraction of wild type cryo-tomograms where zigzag nanodomains were unambiguously visible, proofing the complete lack of nanodomains with statistical significance in a mutant lacking the entire CatSper seemed not ideal. By contrast, *Efcab9*^−/−^ sperm assemble the CatSper complex missing only EFCAB9 and CATSPERζ, the two interdependent non-TM cytosolic subunits that work interdependently as a gatekeeper of CatSper channel^12^ (also see below). Previous observations by super-resolution light microscopy and scanning EM demonstrated a discontinuous and fragmented linearity pattern of the CatSper nanodomains in *Catsperz*^−/−^ *or Efcab9*^−/−^ sperm^8,12^. Therefore, we performed cryo-ET of *Efcab9*^−/−^ sperm flagella, expecting to visualize structural changes reminiscent of the previously described phenotype. Indeed, we observed that the particles positioned to the corresponding locations in the flagellar membrane of *Efcab9*^−/−^ sperm form discontinuous rows, short clusters, or individual repeat units (Fig. 2i–t, Supplementary Fig. 1a, b). Together with the position of these particles along flagella, this genetic evidence, i.e., disruption of the zigzag-rows in *Efcab9*^−/−^ sperm, strongly supports that these particles are macromolecular CatSper channel complexes that form the quadrilinear nanodomains.

Cryo-tomograms of human sperm flagella revealed similar linear rows that are ~24 nm wide and consist of repeating units that are also arranged in a zigzag pattern (Fig. 2u–x), suggesting that assembly of the higher-order CatSper complex is conserved in mammalian sperm. It is of note that a similar zigzag arrangement of particles was previously reported in freeze-fracture EM micrographs of guinea pig and hamster sperm flagella, termed “flagellar zipper”, but in a singular row above the microtubule doublet number 1^21,22^. Given that the CatSper channels form four nanodomains in mouse and human sperm laterally on each side of the two longitudinal columns of the fibrous sheath^8,14,16^ (Figs. 1 and 2), it remains to be determined whether the flagellar zippers in these rodent species are related to the CatSper rows.

### EFCAB9-CATSPERζ complex has a profound impact on the long- and short-range architecture of CatSper channels

In 83 cryo-tomograms collected from cryo-FIB lamella of *Efcab9*^−/−^ sperm flagella, CatSper complexes were visible in 30 tomograms (Supplementary Table 2). Most of these complexes were scattered individual particles or short clusters containing only 1–18 units (Fig. 2k–p), and very few assembled into continuous rows with a maximum of ~70 repeats (Fig. 2q–t, Supplementary Fig. 1a, b). Interestingly, the short mutant clusters are no longer well-aligned with the flagellar axis, i.e., they adopt various angles—up to almost perpendicular—relative to the longitudinal axis of the flagellum (Fig. 2n, p, t). In addition, we observed that the zigzag pattern of CatSper complexes was disrupted in absence of EFCAB9. Instead of the zigzag arrangement observed in wild type sperm, neighboring complexes in *Efcab9*^−/−^ sperm form short arrays of diagonal stripes exhibiting either a backslash (\) or a forward slash (/) array (Fig. 2l, l’, r, r’, Supplementary Movie 2). Our previous study has shown that EFCAB9 is functionally and dependently paired with CATSPERζ^12^ (also see below). Taken all together, these results suggest that the absence of the EFCAB9-CATSPERζ complex from the intracellular side of the channel causes disruption of the higher-order arrangement of the CatSper channel complex and the linear alignment within the longitudinal nanodomains.

### Extracellular structures of CatSper form canopy tents that connect pore-forming channels as beads on a zigzag string

**Fig. 3:**
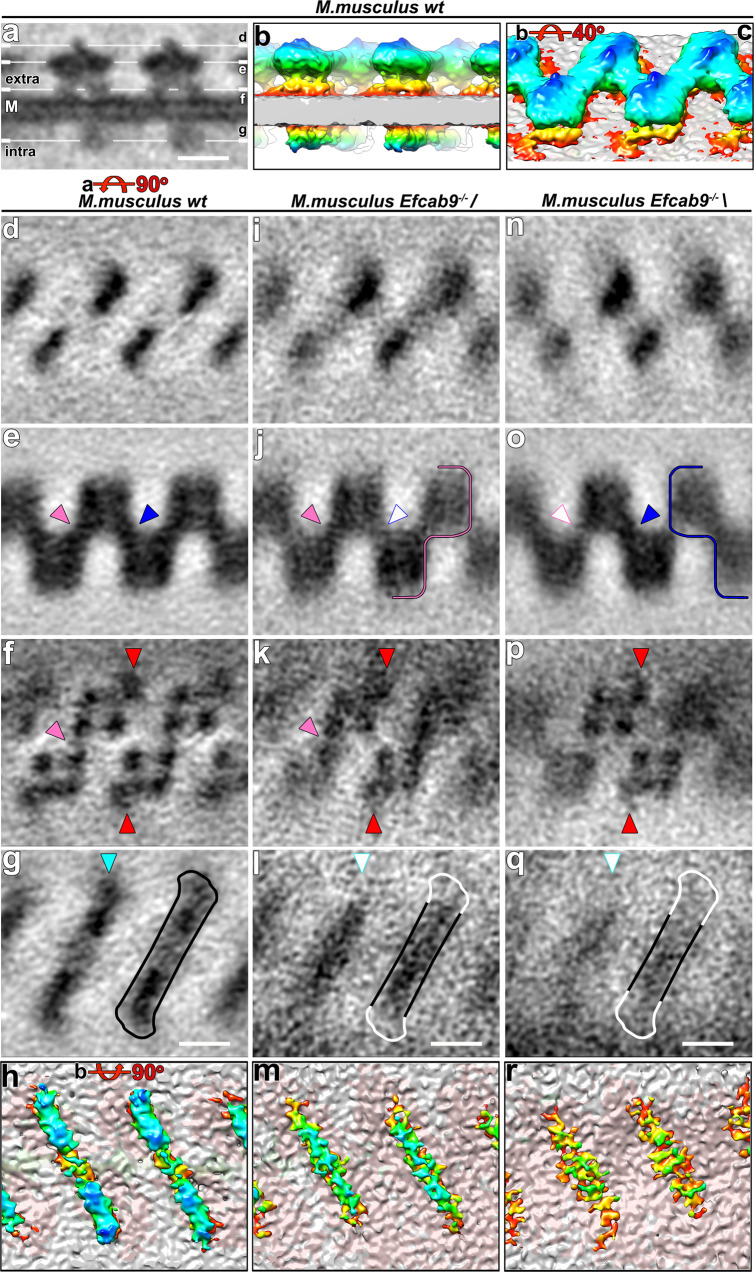
Structural features of the 3D averaged CatSper complex. **a** A tomographic slice showing the side view of averaged CatSper complex structure in wild type mouse sperm. M, membrane. **b**, **c** 3D isosurface renderings of the averaged CatSper complex in wild type mouse sperm: (**b**) side view; (**c**) extracellular domain. **d**–**g**, **i**–**l**, **n**–**q** Tomographic slices showing the averaged CatSper complex structure of wild type (**d**–**g**) and *Efcab9*^−/−^ (/, **i**–**l**; \, **n**–**q**) mouse sperm in top view. The positions are indicated by lines in (**a**) showing the following structural features (roof ridge: **d**, **i**, **n**; canopy roof: **e**, **j**, **o**; tetrameric channel pore: **f**, **k**, **p**; intracellular domain: **g**, **l**, **q**). The filled pink and blue arrowheads in (**e**, **j**, **o**) indicate the presence of electron density between adjacent CatSper complexes, which are weakened in *Efcab9*^−/−^ as indicated by white arrowheads in (**j**, **o**). In (**f**, **k**, **p**) red arrowheads indicate the wing structures, and pink arrowheads highlight an inner connection close to the channel subunits. The filled cyan arrowhead in (**g**) indicates the position of the EFCAB9-CATSPERζ subcomplex, which is missing in *Efcab9*^−/−^ flagella (white arrowheads in **l** and **q**). **h**, **m**, **r** 3D isosurface renderings show bottom views of the averaged CatSper intracellular domain in wild type (**h**) and *Efcab9*^−/−^ (/, **m**; \, **r**) flagella. Scale bars, 10 nm.

After determining the periodicity of the CatSper complexes within the zigzag rows, we performed subtomogram averaging of the repeating units to increase the signal-to-noise ratio and thus the resolution. We averaged ~2500 CatSper complex repeat units (which includes the application of two-fold symmetry) from continuous rows in 11 acquired cryo-electron tomograms of both whole cells and cryo-FIB milled mouse wild type flagella (Supplementary Table 2). The averages depict molecular details of CatSper complexes in situ (Fig. 3a–h) with up to 26 Å resolution (0.5 FSC criterion; or 17 Å using the 0.143 FSC criterion; Supplementary Fig. 1f, Supplementary Table 2).

As shown in Fig. 3 and Supplementary Movie 3, the averaged 3D structure of the zigzag row reveals that the CatSper complexes are evenly spaced in two anti-parallel lines, i.e., the complexes are 180° rotated between the two lines. The appearance of an ~25 nm-wide zigzag-pattern results from the staggering of the rows of channels and the ECDs connecting across the lines (Fig. 3c, e, f). Several structural features of the whole channel unit are visualized from extra- to intracellular domains across the outer and inner leaflet of the membrane bilayer (Fig. 3a–h). In the side view (Fig. 3a), the most prominent structural feature of each CatSper complex is the uniquely shaped ECDs that form a 11.2 nm high canopy tent in which the majority of the ECD mass forms the canopy roof (Fig. 3a–c). The roof is connected between neighboring complexes (Fig. 3e, pink and blue arrowheads) to a continuous zigzag ribbon around 6.6 nm away from the flagellar membrane (Fig. 3a, c).

Tangential slices (i.e., top views) through this extracellular part, clearly show the asymmetric unit closest to the membrane: the tetrameric arrangement of the CATSPER1-4 subunits with an additional density that we named “wing” at the outside corners (Fig. 3f, Supplementary Movie 3, red arrowheads). At an inside corner of the tetramer—opposite to the wing-connected subunit—a fine but clear connection (Fig. 3f, pink arrowhead) could be visualized between the two “forward slash” neighboring channels, suggesting dimer formation. As the position of the wing clearly reveals a 180° rotation, the intra-dimer connection is between the identical subunits of the neighboring CatSper monomers. The diameter of the tetrameric channel is 10.6 nm, which is in a similar range with the size observed for other tetrameric channels such as the 10 nm wide Ca_v_1.1^23^. The center-to-center spacing between channels along the zigzag string is 15 nm.

The ECD canopy roof is positioned right above the tetrameric channel (i.e., the four tent poles) (Fig. 3a, d, e, f). Interestingly, the roof ridge (Fig. 3c, Supplementary Movie 3, dark blue) is off-center and tilted in the same “forward slash” direction toward the wing-side outer pore-forming subunit. Based on the subtomogram average, the mass estimation of the ECDs of one CatSper channel complex is ~450 kDa, close to the sum of the ECDs predicted for the eight known TM subunits (CATSPER1-4, β, γ, δ, and ε) with 1:1 stoichiometry (Supplementary Table 1). We speculated that each TM auxiliary subunit specifically pairs with a particular pore-forming subunit^19^. While this manuscript was under review, Lin et al. (2021) reported the single particle cryo-EM structure of the isolated CatSper complex from mouse testis and epididymis, confirming the predicted stoichiometry and organization within the CatSper monomer^18^.

### Intracellular structures of CatSper connect two channel units as diagonal arrays

Markedly, the intracellular domains form a continuous diagonal array underneath each CatSper dimer with bulges at both ends of the diagonal array (Fig. 3g, h). The diagonal stripes are spaced by 17.6 nm and are oriented in the same forward slash direction as the two connected subunits within a dimer (Fig. 3f, g, h), further supporting that the zigzag rows are formed by interconnected CatSper dimers as building blocks. The side view of the complex shows that the intracellular protrusion of an individual channel is not coaxial with the center of the tetrameric channel (Fig. 3a).

### The effect of EFCAB9-CATSPERζ absence on the intra- and extracellular architecture of the CatSper channel and higher-order assembly

The scattered and short clusters of the CatSper channel complexes and the disrupted zigzag arrangement in *Efcab9*^−/−^ sperm (Fig. 2) suggest that the higher-order assembly of the channels is disrupted in the absence of EFCAB9-CATSPERζ. We thus investigated the structural difference of CatSper channels in *Efcab9*^−/−^ sperm. The repeat units (~500 particles of /; ~1000 of \) were averaged from 19 tomograms of both whole cell samples and cryo-FIB milled lamella of sperm flagella (Supplementary Table 2). To ensure that only CatSper complexes were included during the averaging process, we picked particles only from assemblies containing 3+ subunits in a zigzag arrangement, and excluded any scattered individual particles as these could not be unambiguously identified as CatSper complex. The mutant averages showed that complexes overall are still arranged in two staggered and anti-parallel lines, as is evident from the preserved locations of the tilted roof ridge (Fig. 3i, n, Supplementary Fig. 1d, e) and the wing density (Fig. 3k, p, red arrowheads). The width and ECD mass of the zigzag string in the mutant is comparable with those in wild type (Supplementary Fig. 1c–e). However, in the *Efcab9*^−/−^ mutant the usual zigzag pattern was slightly disrupted. Consistent with the forward slash (/) and backslash (\) arrays observed in tomographic slices of mutant flagella (Fig. 2l, l’, r, r’, Supplementary Movie 2), subtomogram averaging of all picked mutant particles (i.e., forward slash and backslash arrays together) followed by classification, automatically separated the mutant particles into two class-averages: in the forward slash conformation where the dimer-connection appears wild type-like (Fig. 3j, pink arrowhead), whereas the inter-dimer connection seems weakened compared to the wild type average (Fig. 3j, white arrowhead), which is vice versa in the backslash conformation, i.e., weaker intra-dimer and wild type-like inter-dimer connection (Fig. 3o, white and blue arrowheads, respectively). The forward slash and backslash conformations can form (short) arrays with themselves, but do not seem to mix within the same CatSper-cluster. This apparent interaction incompatibility between the conformations and the weakening of inter- or intra-dimer interactions, respectively, likely causes the observed fragmentation of the CatSper rows in the *Efcab9*^−/−^ mutant (Supplementary Fig. 5a). Strikingly, the intracellular EM density of the CatSper dimer lacks the bulges at both ends of the diagonal stripe in both mutant class averages (Fig. 3g, h vs. 3l, m, q, r), indicating the likely location of the EFCAB9-CATSPERζ complex.

### Similarities and differences between mouse and human sperm CatSper structures

Cryo-tomograms and subtomogram average of human sperm flagella also revealed an ~24 nm wide zigzag row of staggered complexes on the extracellular side of the flagellar membrane (Fig. 2u–x, Supplementary Fig. 1i–l). Although the resolution of the subtomogram average of human sperm flagella was limited by a low number of averaged repeats (Supplementary Fig. 1h, Supplementary Table 2), a 11.3 nm wide tetrameric channel and a center-to-center spacing between channels along the zigzag string of 15.2 nm (Supplementary Fig. 1k, Supplementary Fig. 5b) together with the canopy roofs in a zigzag-pattern and the intracellular diagonal array connecting two channel units (Supplementary Fig. 1j, l) were clearly visible. The findings suggest that these unique zigzag rows (Fig. 2u–x) are arrays of human CatSper complexes in the presumably anti-parallel arrangement like mouse CatSper. To fairly compare similarities and differences, the higher-resolution average structure of wild type mouse CatSper was low-pass filtered to 7.9 nm, i.e., to the same resolution as that of the averaged structure human CatSper (Supplementary Fig. 1i–p). We observed a few differences between the mouse and human sperm CatSper complex arrays: first, the human tetrameric channels appear slightly rotated relative to the mouse channel, meaning in human CatSper only the two subunits that form the dimer-connection are closest to the center of the zigzag row, whereas in mouse the intra- and inter-dimer forming channel subunits are aligned next to each other in the center of the zigzag row (Supplementary Figs. 1k vs. 1o, and 5b); this rotation may contribute to the seemingly opposite orientation of the ECD roof ridges (Supplementary Fig. 1i vs. 1m); second, human CatSper appears to be missing the wing structure which is clearly visible beside the mouse CatSper tetrameric channel at both resolutions (compare Supplementary Fig. 1k vs. 1o; Supplementary Fig. 5b), suggesting the presence of a species-specific component of the CatSper complex in murine sperm.

### Molecular identities of the wing structure and the intracellular dimer-array

We investigated whether the mouse-specific wing structure and the intracellular diagonal array bridging two channel units within a CatSper dimer could be explained at the molecular levels. Ten components have been validated to comprise the CatSper channel complex in the linear nanodomains^6^. However, we previously showed by comparative mass-spectrometry that in mouse *Catsper1*^−/−^ sperm—that lack the entire CatSper channel complex—four additional proteins were significantly reduced: DNA-binding ATPase FANCM (Fanconi anemia, complementation group M), C2CD6 (C2 Calcium-dependent Domain-containing protein 6, also known as ALS2CR11), SLCO6C1 (Solute carrier organic anion transporter family, member 6c1), and E3 ubiquitin-protein ligase TRIM69 (Tripartitle motif containing 69), suggesting these proteins are CatSper-associated candidates^12^ (see also Supplementary Fig. 2a).

**Fig. 4:**
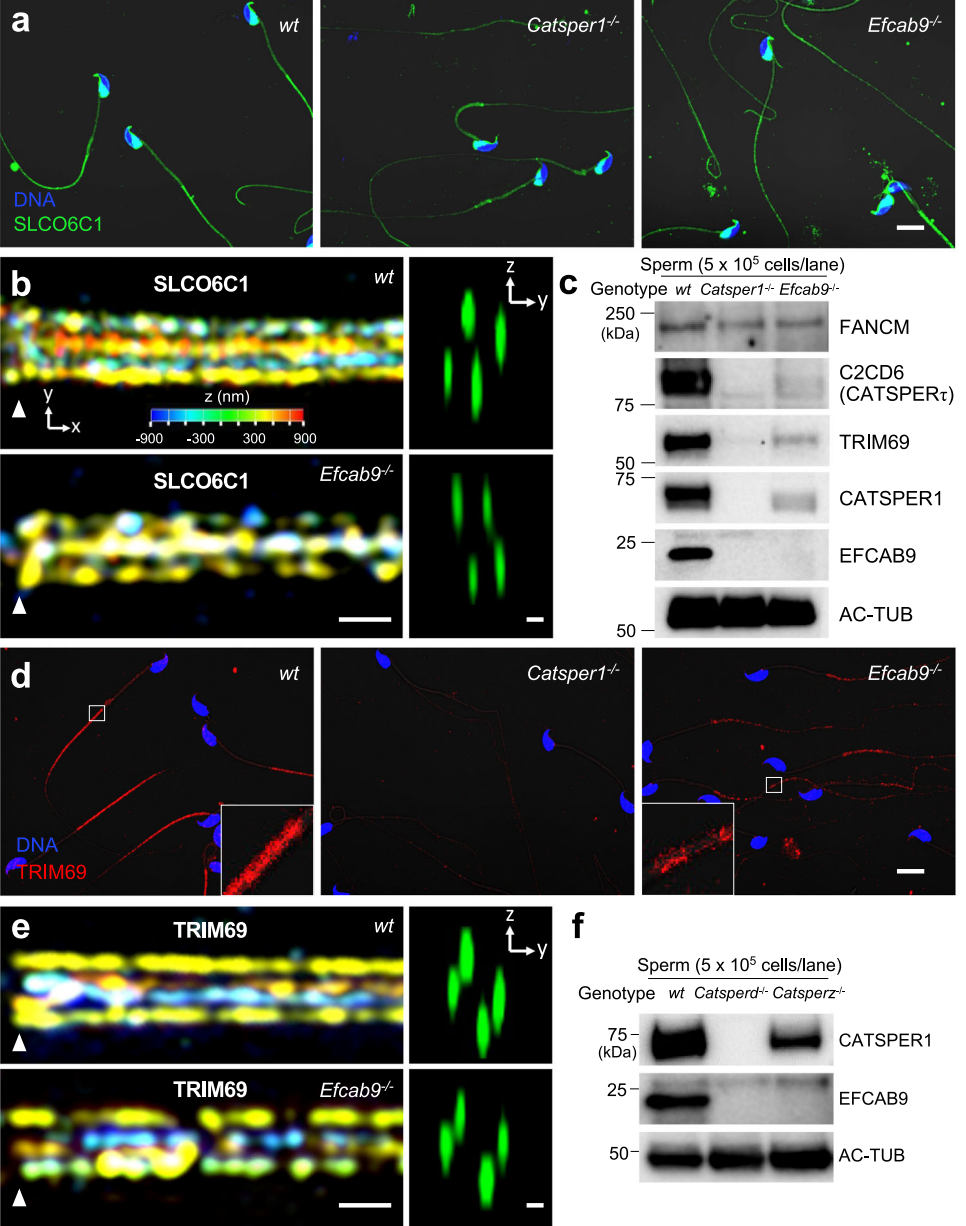
Subcellular localization and protein levels of candidate CatSper-associated proteins in mouse sperm. **a** Immunolocalization of SLCO6C1 in mouse wild type, *Catsper1*^−/−^ and *Efcab9*^−/−^ sperm by confocal light microscopy. Sperm heads are stained blue using Hoechst. **b** Quadrilinear arrangements of SLCO6C1 visualized by 3D structured illumination microscopy (SIM) in sperm from wild type (upper) and *Efcab9*^−/−^ (lower) mice. Colors in *xy* projections encode the relative distance from the focal plane along the *z* axis. Arrowheads indicate the annulus, i.e., the junction between the midpiece and principal piece of the sperm tail. *yz* projections (cross sections) are shown on the right. **c** Western blot analyses of three candidate CatSper-associated proteins, FANCM, C2CD6 and TRIM69, in wild type and two CatSper mutants. **d** Immunolocalization of TRIM69 in mouse wild type, *Catsper1*^−/−^ and *Efcab9*^−/−^ sperm by confocal light microscopy. Sperm heads are stained blue using Hoechst. **e** Quadrilinear arrangements of TRIM69 visualized by 3D SIM in sperm from wild type (upper) and *Efcab9*^−/−^ (lower) mice. **f** Western blot analyses of EFCAB9 in wild type, *Catsperd*^−/−^ and *Catsperz*^−/−^ sperm. Scale bars, 10 μm in (**a** and **d**); 500 nm in *xy* projection (left) and 200 nm in *yz* projections (right) of (**b** and **e**). All shown images are representative images from three biological and technical replicates (*n* = 3).

Among these candidates, we proposed that the wing structure—obviously seen extracellularly (Fig. 3f, red arrowheads) next to the tetrameric channel—might consist of SLCO6C1, because it is not only a rodent-specific but also multi-pass TM protein, whereas FANCM, C2CD6 and TRIM69 are cytoplasmic proteins conserved in both mice and humans^12,15,24^. We generated a SLCO6C1 antibody that was able to detect recombinant SLCO6C1 by Western immunoblots (Supplementary Fig. 2b), and both recombinant and native SLCO6C1 in mouse sperm by fluorescence light microscopy (under non-denaturing conditions) (Fig. 4a, Supplementary Fig. 2c, d). SLCO6C1 is localized in the sperm principal piece, the longest part of the sperm flagellum harboring the CatSper channel in high concentration (Fig. 4a). Using 3D structured illumination microscopy (3D SIM), we revealed that SLCO6C1 is quadrilinearly distributed along wild type mouse sperm flagella, which also becomes more discontinuous in the absence of EFCAB9 (Fig. 4b). Both the localization pattern in four nanodomains and the fragmentation in the *Efcab9*^−/−^ mutant sperm is the same phenotype previously shown for other known CatSper TM subunits^6^. A recent, independent study by Lin et al. (2021) also identified SLCO6C1 by mass spectrometry (MS) analysis of the isolated mouse CatSper channel complex and modeled it into their cryo-EM reconstruction of isolated monomeric CatSper^18^, validating this intriguing physical association of the ion channel-transporter complexes. Physiological substrates are not yet identified for SLCO6C1^25^. The International Mouse Phenotyping Consortium reports that *Slco6c1*^−/−^ mice are fertile^26^, suggesting that SLCO6C1 function is not essential but modulatory for regulating CatSper and sperm motility. CatSper is required to sustain motility for an extended period^7,12^, which is dependent on flagellar energy metabolism^27,28^. As the wing structure is seen in the mouse but not human CatSper (Supplementary Fig. 1), the association of SLCO6C1 with CatSper channel complex might be a species-specific molecular mechanism such as linking Ca^2+^ homeostasis to ATP production. Compared with mouse sperm, which use glycolysis as a dominant source of ATP production^29^, human sperm might split ATP production differently between oxidative phosphorylation and glycolysis. The sperm phenotypes of *Slco6c1*^−/−^ males in detail remain as future areas of investigation.

We next examined the protein levels of FANCM, C2CD6, and TRIM69 in *Catsper1*^−/−^ and *Efcab9*^−/−^ sperm to determine whether these three candidates are truly associated with the CatSper channel. Using western blot analyses, we found that C2CD6 and TRIM69 were barely detected in *Catsper1*^−/−^ and significantly reduced in *Efcab9*^−/−^ sperm, whereas the FANCM protein level was not affected (Fig. 4c), suggesting that only C2CD6 and TRIM69 are candidate CatSper-associated proteins. Moreover, fluorescence light microscopy showed that the two CatSper-dependent proteins, C2CD6 and TRIM69, localize in the principal piece (Fig. 4d, Supplementary Fig. 2f, g)^15^, specifically to four nanodomains along the flagella as seen by 3D SIM imaging of TRIM69 (Fig. 4e). In the absence of EFCAB9-CATSPERζ complex^12^ (Fig. 4f), the TRIM69 distribution is fragmented, resembling previously reported results for other known CatSper subunits^8,12^. Additional recent studies from our and another lab, also support physical association of C2CD6 and TRIM69 with the CatSper complex, including visualizing a quadrilinear distribution of C2CD6 along mouse sperm flagella^15,30^ and showing co-immunoprecipitation of TRIM69 with C2CD6^15^. MS analysis of purified CatSper complexes also identified C2CD6 as one of the top high-confidence proteins^18^. All-in-all, C2CD6 and TRIM69 are likely bona fide CatSper-associated proteins in mammalian sperm. Genetic abrogation of *C2cd6* (now named as *Catspert*) impairs sperm hyperactivation and male fertility^15,30^. By contrast, *Trim69* is not essential for fertility^31,32^, indicating its function on the CatSper channel is likely to be modulatory and/or indirect. We hypothesize that they presumably comprise the middle bar of the intracellular diagonal arrays that remain visible in the subtomogram averages of *Efcab9*^−/−^ CatSper complex (Fig. 3h vs. 3m, r).

### Atomic model fitting to the averaged in situ CatSper complex reveals supramolecular interactions and the molecular basis of the zigzag assembly

**Fig. 5:**
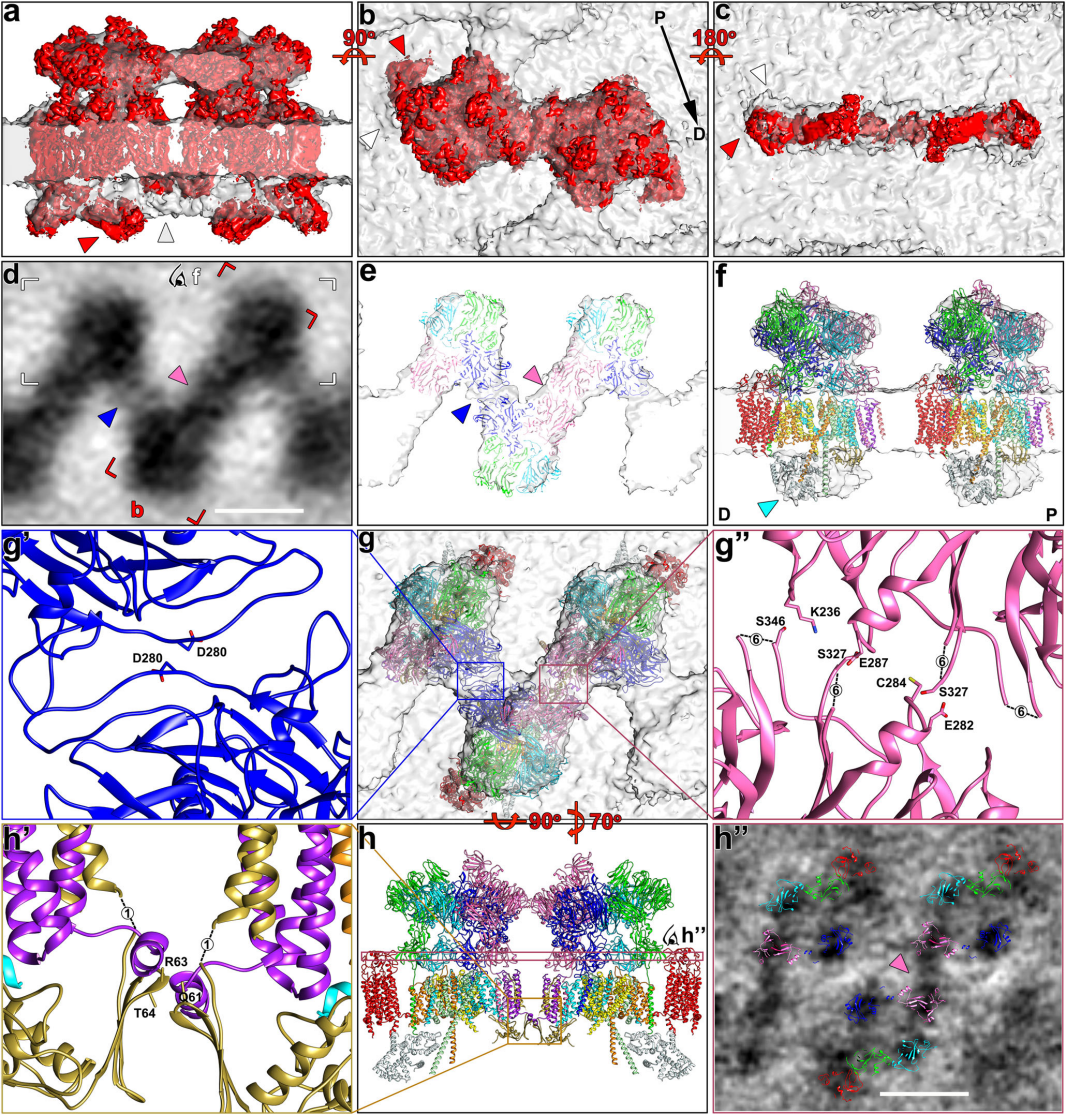
Molecular interactions between CatSper complexes in the in situ assembly. **a**–**c** Two single particle cryo-EM density maps of monomeric CatSper complex^18^ (red) docked into the averaged cryo-ET map of the in situ CatSper complex (gray): (**a**) side view; (**b**) top view; (**c**) bottom view. Red arrowheads indicate several positions where the monomeric CatSper structure^18^ does not fit well within the in situ structure; the gray arrowhead in (**a**) indicates an intracellular structure not observed in the single particle CatSper structure; the white arrowhead in (**b**) indicate the correct position of the wing structure (SLCO6C1) (see also supplementary Fig. 3). The flagellar axis is indicated from proximal (P) to distal (D) in (**b**). **d** A tomographic slice of the averaged CatSper structure showing the connected canopy roof of the zigzag arrangement in wild type mouse sperm. Red box indicates a CatSper complex dimer as shown in (**b**). The white box and eye symbol indicate the view in (**f**). **e**, **f** Atomic models of three CatSper monomers docked into the averaged cryo-ET map of the in situ CatSper complex showing the canopy-forming ECD in top view (**e**) and two channels on the same side of the zigzag row in side view (**f**). As modeled previously^18^, CATSPER1, 2, 3, and 4 are colored in yellow, orange, pale green and cyan, respectively; CATSPERβ, γ, δ and ε are colored in pink, blue, light blue, and green, respectively. SLCO6C1 with corrected position is red colored. CATSPERη and TMEM249 are colored purple and gold, respectively. The intracellular EFCAB9-CATSPERζ subcomplex is colored light cyan. The arrowheads in (**d**, **e**) indicate two interfaces at the canopy level: the dimer interface between two CATSPERβ subunits (pink), and the inter-dimer interface between two CATSPERγ subunits (blue). The cyan arrowhead in (**f**) indicates the EFCAB9-CATSPERζ subcomplex; note that proximal (P) is on the right and distal (D) of the flagellum on the left in (**f**). **g**–**g**” Top view of the atomic model of three CatSper complexes in ribbon presentation docked into the averaged cryo-ET map (**g**) to show potential interactions between two CATSPERγ (**g**’), and two CATSPERβ subunits (**g**”) at the interface between dimers (**g**’) and at the dimer interface (**g**”), respectively. **h**–**h**” Side view of the pseudo-atomic model of a CatSper complex dimer (**h**) showing potential intracellular interactions between two TMEM249 subunits (**h**’). A tomographic slice through the ECD near the membrane/channel level (**h**”) shows a connection between two CatSper complexes (pink arrowhead) at the dimer interface, but the atomic model of the monomeric complex lacks structures in the corresponding position. Predicted interacting amino acids are indicated in (**g**’, **g**” and **h**’); dash lines indicate unresolved amino acids in the atomic model^18^ with amount of missing residues labeled. Scale bars: 10 nm in (**d** and **h**)”.

The single particle cryo-EM structure of isolated, monomeric CatSper complex linked to SLCO6C1 (CatSpermasome) has recently become available^18^, enabling us to fit this high-resolution structure into our subtomogram average of the cellular higher-order arrangement of CatSper (Fig. 5, Supplementary Movie 4). The authors of the single particle cryo-EM study used a combination of de novo model building, homology modeling and docking of structure predictions to generate a (pseudo-)atomic model of most of the CatSper subunits in the monomer^18^. Using the *fit-in-map* tool of Chimera, we were able to fit the cryo-EM density and corresponding atomic model of two CatSper monomers (Fig. 5a–c, red) into a dimer of the cryo-ET map (Fig. 5a–c, gray) with high confidence (correlation score 0.8) (Fig. 5a–c, f, g; Supplementary Movie 4). This revealed details of the molecular interactions within and between the CatSper channel dimers, which are critical for the zigzag assembly.

Most of the extracellular regions of the monomeric high-resolution structure and atomic model^18^ fitted very well to the averaged in situ structure (Fig. 5a, b, f, g). This revealed e.g., that the canopy-roof of the in situ structure is mostly formed by the β-propeller domains of the four auxiliary CatSper subunits (CATSPERβ, γ, δ, and ε) (Fig. 5d, e), that the extracellular inter- and intra-dimeric connections between CatSper complexes are formed by pairwise molecular interactions between two CATSPERγ (Fig. 5d, e, g, g’, blue colored subunit; Supplementary Fig. 5a) or two CATSPERβ (Fig. 5d, e, g, g”, h, h”, pink colored subunit; Supplementary Fig. 5a), respectively, and that the tallest auxiliary subunit, CATSPERβ, forms the protruding roof ridge (Fig. 5f; Supplementary Movie 4). However, we found also one major difference between the 3D structures of purified, monomeric^18^ and our assembled in-cell CatSper complex: the position of SLCO6C1, which interacts with the stem domain of CATSPERε (green colored), is slightly rotated and shifted by 3 nm in the direction of CATSPERγ (Fig. 5b, red arrowhead) compared with the “wing” structure in our in situ map (Fig. 5b, white arrowhead). This difference is mostly likely due to a preparation artifact of the purified CatSper complex, which included detergent treatment and cross-linking^18^. Therefore, we fitted the atomic map of SLCO6C1 independently from the remaining CatSper map to our in situ structure (Supplementary Fig. 3) and display the (pseudo)atomic model of the CatSpermasome with SLCO6C1 in the corrected position (Fig. 3f–h, red colored subunit).

In contrast to the extracellular regions, the intracellular (cytosolic) regions of the monomeric high-resolution structure^18^ did not fit as well into the averaged in situ structure (Fig. 5a, c). Specifically, we observed the following major differences: first, in the bottom view it is evident that after rigid-body fitting (with the large ECD fitted well) the monomeric EM density is rotated a few degrees out of the in situ structure (Fig. 5c, compare red and white arrowheads); second, the side view shows that the single particle EM density termed “cytosolic map 1” by Lin et al^18^ clearly projects out of the in situ structure away from the membrane (Fig. 5a, red arrowhead), leaving the central part of the intracellular diagonal array of the in situ structure unoccupied, including the intracellular dimer interface (Fig. 5a, light gray arrowhead). In the single particle cryo-EM study, the authors reported that the cytosolic (intracellular) region of purified CatSper was flexible and resulted in a relatively low resolution reconstruction (6–10 Å), allowing only fitting of predicted structures of the EFCAB9-CATSPERζ subcomplex into the “cytosolic map 2”, and leaving the “cytosolic map 1” unidentified^18^. Comparison to the in situ structure that shows similar resolution of the extra- and intracellular regions, suggests that the structural flexibility and position of “cytosolic map 1” far away from the membrane is most likely a preparation artifact of the purified monomeric CatSper complex that lacks the dimer-interactions and native membrane. Placement of the pseudo-atomic model of the EFCAB9-CATSPERζ subcomplex in the “cytosolic map 2” below CATSPER2/CATSPERε^18^, which corresponds to the bulges at the ends of the diagonal intracellular domain in the in situ structure (Fig. 5a, f, h), is consistent with the cryo-ET comparison between wild type and *Efcab9*^−/−^ CatSper complex, i.e., the bulges were missing in the mutant averages (compare Fig. 3g, h to 3l, m, q, r). C2CD6 and TRIM69 might contribute to the central part of the intracellular regions in the in situ structure, but at this point, stoichiometry of these and other subunits remains to be determined, and interaction of the cytosolic proteins C2CD6 and TRIM69 with CatSper might also be transient and thus difficult to resolve by cryo-EM.

To further understand the key interactions that might drive stable assembly of the zigzag row of CatSper complexes in situ, we examined the molecular interactions between neighboring CatSper complexes as predicted by the fitting of the atomic model to the in situ structure (Fig. 5d–h). At least three main interfaces with potential molecular interactions were identified, two between ECD and one in the intracellular region. We found that within a dimer, multiple amino acids (e.g., K236, E282, C284, E287, S327 and S346) of the β-propeller (roof) domain of two CATSPERβ subunits may interact (Fig. 5e, g, g”, h, h”, pink colored subunit). Intracellularly, TMEM249—a newly identified TM protein of the CatSpermasome^18^—exhibits several potential interactions at the dimeric interface (e.g., Q61, R63 and T64) (Fig. 5h, h’, gold colored subunit). Between dimers, β-loops in the extracellular roof region of CATSPERγ (e.g., D280) seem to interact (Fig. 5e, g, g’, blue colored subunit). However, considering that some residues near the ECD interfaces and large parts of the intracellular regions were not resolved in the single particle cryo-EM structure and thus are not included in the atomic model^18^, additional molecular interactions between CatSper complexes in situ are possible. For example, a connecting EM density is clearly visible in the in situ structure at the extracellular dimeric interface close to the membrane (Fig. 3f, k, pink arrowheads), but the single particle reconstruction and atomic model of the monomer channel lack density and molecular interactions in the corresponding region (Fig. 5a, h, h”). Considering that none of the newly identified TM subunits (CATSPERη, TMEM249, and an unassigned (gray) helix) show large ECDs, the present monomeric structure and atomic model of the CatSpermasome could be missing a (TM) component that remains to be identified. Our pseudo-atomic model of the higher-order assembly of CatSper provides a foundation for future experiments to identify this missing link at the dimer interface, e.g., by targeted cross-linking or proximity labels that probe vicinity interactomes around known sites.

We previously showed that the EFCAB9-CATSPERζ subcomplex has (at least) two functions in the CatSper: regulating the pH-dependent activation and Ca^2+^ sensitivity of the channel and maintaining the connectivity of the nanodomains^12^. Here, our data show that the conformation of the CatSper channel ECD is altered in the absence of the intracellular EFCAB9-CATSPERζ subcomplex (Fig. 3), directly linking the roles of this subcomplex to the structural assembly and regulation of the CatSper. Disruption of the intracellular domain seems to cause a “distortion” of the monomer with two possible states: one with a weakened intra-dimer interaction (\) due to the lack of interaction between CATSPERβ of neighboring complexes (Fig. 5h’), and the other with a weakened inter-dimer interaction (/) due to two CATSPERγ of neighboring complexes being pulled further apart (Supplementary Fig. 5a). These two states do not seem to be mixed within the same (short) CatSper mutant clusters and thus do not appear to be compatible to form the zigzag assembly, resulting in the fragmentation of the CatSper rows in the mutant (Fig. 2, Supplementary Fig. 5a). As the proper intracellular interactions not only drive dimer formation but also affects the extracellular connections in the higher-ordered organization of the channel, ECD might be responsible for cooperative channel activity for efficient Ca^2+^ signal transduction along the 50 (human) and 120 (mouse) µm long sperm tails. It will be interesting to study in the future if the opening of the CatSper channels during sperm capacitation, results also in conformational and/or higher-order organizational changes of the CatSper channel complex.

### Structural defects of mutant CatSper correlate with proximally stiff flagellum and compromised motility

**Fig. 6:**
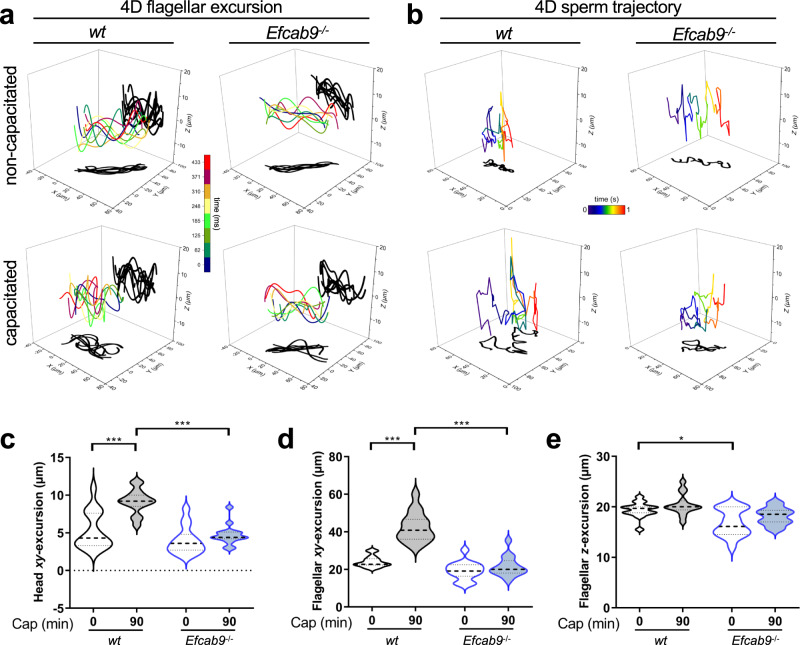
Flagellar beating waveform of free-swimming sperm in four dimensions. **a** 4D flagellar beating waveform analyses of wild type and *Efcab9*^−/−^ sperm by high-speed digital holographic microscopy (DHM). The time-lapse trace of a flagellum at 3D position (laboratory-fixed frame of reference *xyz*) is visualized in color and its projections onto *xy*- and *xz*-planes are shadowed in black. **b** 4D sperm trajectory analyses of wild type and *Efcab9*^−/−^ sperm by DHM. The swimming trajectory of sperm is visualized by tracing the head position. **c**–**e** Statistical analyses of head (**c**) and flagellar (**d**) *xy*-excursion, and flagellar *z*-excursion (**e**) from (**a**). *n* = 15 per group of three animals, a two-way repeated-measures ANOVA statistical test was performed, *p (0.0177) < 0.05, ***p < 0.001, *p* value below 0.001 cannot be given as exact value; the medians (thick dash lines) and interquartile ranges (thin dash lines) are indicated.

To better understand how the here observed structural changes of the mutant CatSper channel complexes may translate into altered flagellar curvature and motility in *Efcab9*^−/−^ sperm, we characterized the flagellar waveform and swim paths of free-swimming sperm in detail over time using 3D high-speed Digital Holographic Microscopy^33^ (Fig. 6, Supplementary Fig. 4). The CatSper-mediated increase in intracellular Ca^2+^ is important for triggering hyperactivated motility during capacitation^34–36^. During this process, wild type mammalian sperm dramatically increased the *xy*-displacement of their flagellum, i.e., in the out-of-plane beating direction (Fig. 6a, d, Supplementary Fig. 4a). In contrast to the increased *xy*-displacement of capacitated wild type sperm flagella, we show here, that capacitation does not significantly affect the flagellar *z*-displacement, i.e., the waveform amplitude, of wild type sperm (Fig. 6d, e, wild type; compare 0 vs. 90 min; Supplementary Fig. 4a). This suggests that Ca^2+^ influx by CatSper activation during capacitation mainly triggers asymmetric out-of-plane beating in the *xy*-direction, but not flagellar movement in the *z*-direction.

For the motility of *Efcab9*^−/−^ mutant sperm, we found that the typical increase in the *xy*-displacement during capacitation is abolished in the mutant (Fig. 6a, c, d, Supplementary Fig. 4a), i.e., head and flagellar *xy-*displacements remain at the same level as that of non-capacitated mutant sperm. This causes mutant sperm to swim inefficiently with lower rotational torque than wild type sperm (Supplementary Fig. 5c). We also found that the *z*-displacement of non-capacitated *Efcab9*^−/−^ sperm is smaller than that of non-capacitated wild type sperm (Fig. 6a, e, Supplementary Fig. 4a). Our previous flagellar waveform analyses of head-tethered sperm showed that the absence of the EFCAB9-CATSPERζ subcomplex caused stiff flagella in the proximal region, which constrains the motion of mutant sperm to that of a propeller-driven rod^8,12^ (Supplementary Fig. 5c). Thus, the here measured reduction in *z*-displacement of non-capacitated *Efcab9*^−/−^ sperm is likely due to this proximal stiffening of the flagellum (see also Supplementary Fig. 5c).

To further dissect the effects of altered flagellar beating patterns on the swimming path of the sperm cells, we measured the 3D trajectories of free-swimming sperm by tracing the head positions using 3D high-speed Digital Holographic Microscopy. Consistent with the increase of asymmetry in beating during capacitation (Supplementary Fig. 4b), the swimming paths of capacitated wild type sperm tracked by their head location showed an increased range of excursion in all dimensions (Fig. 6b, left; compare Supplementary Movie 5 vs. 6). This increased spatial sampling of sperm trajectories during capacitation is believed to be important for effective sperm navigation, enabling sperm to change directions and increasing torque for egg fertilization. In contrast to wild type, we observed that capacitated *Efcab9*^−/−^ sperm fail to expand the 3D excursion range (Fig. 6b, right). This is likely caused by the lack of *xy*-displacement response during capacitation, and highlights the importance of the intact higher-order assembly of CatSper complex for orchestrating CatSper channel activity and thus effective sperm navigation during capacitation. In addition, we have previously shown that wild type sperm exhibit a strict clockwise swimming path^33^, whereas this chirality is randomized in *Efcab9*^−/−^ sperm^37^. Based on our structural findings and the motility defects in *Efcab9*^−/−^ sperm, we propose that in wild type sperm the connected higher-order zigzag arrangement within a longitudinal nanodomain could allow for coordinated opening of the entire array of CatSper channels along the flagellar axis, ensuring a large and synchronized Ca^2+^ influx to generate strong bending force (Supplementary Fig. 5c). In contrast, disruption of the CatSper zigzag-rows and misalignment of short CatSper clusters from the longitudinal axis would dysregulate this domino-effect of Ca^2+^ entry, and thus would impair maintenance of homeostasis and an efficient propagation of the intracellular Ca^2+^ wave^35,36,38^, resulting in a proximally stiff flagellum and altered sperm motility (Supplementary Fig. 5c).

Our study of the higher-order structure of CatSper complex in situ under non-capacitated conditions, forms the foundation for future structural studies of capacitated sperm flagella, to better understand the structural changes that both individual CatSper channels (e.g., open vs. closed state), and the supramolecular interactions and quaternary structures of the CatSper complexes undergo during capacitation. Taken together, this work provides new insights into the structural basis and molecular mechanisms of CatSper channel assembly and regulation of mammalian sperm motility.

## Methods

### Human subjects

A total of three healthy volunteers aged 25–39 were recruited and consented to participate in this study. Freshly ejaculated semen samples were obtained by masturbation and spermatozoa purified by the swim-up technique at 37 °C as described^39^. All processed samples were normozoospermic with a cell count of at least 3 × 10^7^ sperm cells per mL. The experimental procedures utilizing human-derived samples were approved by the Committee on Human Research at the University of California, Berkeley, IRB protocol number 2013-06-5395.

### Animals

*Catsper1*^−/−^, *Catsperd*^−/−^, *Catsperz*^−/−^ and *Efcab9*^−/−^ mice generated in the previous studies^5,8,9,12^ are maintained on a C57/BL6 background. Mice were housed at a temperature of 20–25 °C under a 12/12 h light/dark schedule with 2–5 mice per cage and treated in accordance with guidelines approved by the Institutional Animal Care and Use Committees (IACUC) of Yale University (#20079).

### Mouse and human sperm preparation for biochemical study

Epididymal spermatozoa from adult male mice (8–12 weeks old) were collected by swim-out from caudal epididymis in M2 medium (EMD Millipore). To induce capacitation, collected sperm were incubated in human tubular fluid medium (EMD Millipore) at 2 × 10^6^ cells/ml concentration at 37 °C, 5% CO_2_ condition for 90 min.

Frozen vials of human sperm from healthy, normal male donors were purchased (Fairfax Cryobank). Vials were thawed and mixed with pre-warmed HEPES-buffered saline (HS)^8^ containing (in mM): 135 NaCl, 5 KCl, 1 MgSO_4_, 2 CaCl_2_, 20 HEPES, 5 D-glucose, 10 Lactic acid, 1 Na pyruvate, pH 7.4 adjusted with NaOH, osmolarity 320 mOsm/L. After being washed twice, sperm were placed on top of 20% Percoll (Sigma Aldrich) in HS and incubated at 37 °C for 30 min to allow motile sperm to swim-into the Percoll layer. After removing the top layer containing immotile fraction, sperm cells with high motility were collected by centrifugation at 2000 *g* and resuspended in HS.

### Primary antibodies

Rabbit polyclonal antibodies specific to mouse CATSPER1^5^, EFCAB9^12^, and C2CD6^15^ were described previously. Briefly, to produce antibodies, peptides corresponding to mouse SLCO6C1 (1-14, MAHVRNKKSDDKKA, cysteine added to C-terminus) (GenScript) were synthesized and conjugated to KLH carrier protein. Antisera from the immunized rabbits were affinity-purified using the peptide immobilized on Sulfo Link Plus resin (Pierce). Other antibodies used in this study are commercially available as follows: TRIM69, Origene; FANCM, Affinity Biosciences; acetylated tubulin, Sigma; anti-HA (clone 6E2), Cell Signaling Technology. All the chemicals were from Sigma Aldrich unless otherwise indicated.

### Generation of mouse *Slco61* construct to express recombinant SLCO6C1

Mouse *Slco6c1* ORF clone (#OMu06346) purchased from GenScript was used for generating a plasmid encoding N-terminal Flag- and C-terminal HA-tagged mouse SLCO6C1 by using PCR with the following primers (forward: 5′-TCGACTTAACAGATCGCCACCATGGATTACAAGGATGACGACGATAAGGGTTCAGGTGGTATGGCCCATGTCCGC-3′ and reverse: 5′-ACATCGTATGGGTAACCACCTGAACCAGCCTTTCTTTCTTTTTGTTCTTTTAC-3′). The PCR product was assembled into phCMV using NEBuilder HiFi DNA Assembly (NEB) and referred to as flag-mSLCO6C1-HA in the study.

### Transfection, immunocytochemistry and immunoprecipitation of mSLCO6C1

HEK293T cells were transiently transfected with plasmids encoding untagged mouse SLCO6C1 (mSLCO6C1) or flag-mSLCO6C1-HA, by using Lipofectamine 2000 (Invitrogen). After 24–36 h transfection, cells were harvested for further analyses. Cells were adhered to a glass coverslip that was coated with 0.01% poly-L-lysine, then were washed with PBS twice and fixed with 4% paraformaldehyde (PFA) in PBS for 10 min at RT. The fixed cultured cells were permeabilized with 0.1% Triton X-100 in PBS for 10 min at RT and blocked with 10% normal goat serum in PBS for 1 h at RT. Cells were incubated overnight with primary antibodies at 4 °C. Primary antibodies used for immunocytochemistry were: mouse monoclonal anti-HA (1:100) and rabbit polyclonal mouse SLCO6C1 (10 µg/mL). Immunostained samples were washed with PBS three times, followed by incubation with goat anti-rabbit or mouse IgG conjugated with Alexa 488 or Alexa 568 (1:2,000, Invitrogen) and Hoechst 33342 (5 µg/ml, Invitrogen) in blocking buffer for 1 h. After washing with PBS three times, coverslips were mounted with ProLong^™^ Gold Antifade reagent (Invitrogen) sealed with nail polish. For immunoprecipitation, after 24 h transfection, 1 × 10^6^ cells in a six-well plate were washed with PBS twice. Cell suspensions were pelleted down by centrifugation at 500 *g* for 5 min and cell pellets were lysed with lysis buffer containing 50 mM Tris-HCl, 150 mM NaCl, 5% glycerol, 1% TritonX-100, and 1X EDTA-free protease inhibitor (Roche) by rocking at 4 °C for 1 h, followed by centrifugation at 14,000 *g* for 1 h at 4 °C. 20% solubilized protein in supernatant were preserved as input control and 80% solubilized protein fractions were further mixed with 2 µL of HA magnetic beads (clone 2-2.2.14, Pierce) by rocking at 4 °C for overnight. HA magnetic bead mixtures were washed with 0.1% Triton X-100 for three times. HA magnetic bead mixtures and input samples were eluted and/or denatured with 2X LDS sampling buffer and 50 µM dithiothreitol (DTT) at 75 °C for 10 min.

### Western blot analysis

Whole sperm protein content was extracted as previously described^8,9,14^. In short, mouse epididymal or human spermatozoa washed in PBS were directly lysed in a 2X SDS sample buffer. The whole sperm lysate was centrifuged at 15,000 *g*, 4 °C for 10 min. After adjusting DTT to 50 mM, the supernatant was denatured at 95 °C for 10 min before loading to gel. Antibodies used for Western blotting were antibodies against CATSPER1 (1 µg/mL), EFCAB9 (1 µg/mL) and C2CD6 (1 µg/mL), TRIM69 (0.5 µg/mL), SLCO6C1 (1 µg/mL), FANCM (1 µg/mL) and acetylated tubulin (1:10,000). Secondary antibodies were anti-rabbit IgG-HRP (1:10,000), anti-goat IgG-HRP (1:10,000) and anti-mouse IgG-HRP (1:10,000) from Jackson ImmunoResearch (West Grove). Uncropped and unprocessed scans of full blots are provided as a Source Data File.

### Sperm immunocytochemistry

Sperm were washed in PBS twice, attached on glass coverslips, and fixed with 4% PFA in PBS at room temperature (RT) for 10 min (mouse) or at 4 °C for 1 h (human). Fixed samples were permeabilized using 0.1% Triton X-100 in PBS at RT for 10 min, washed in PBS, and blocked with 10% goat serum in PBS at RT for 1 h. Cells were stained with anti-C2CD6 (10 μg/mL), TRIM69 (5 μg/mL) SLCO6C1 (10 μg/mL), in PBS supplemented with 10% donkey serum at 4 °C overnight. After washing in PBS, the samples were incubated with donkey anti-goat Alexa 568 (Invitrogen, 1:1,000) in 10% donkey serum in PBS at RT for 1 h. Hoechst was used to counterstain nuclei for sperm head visualization. Immunostained samples were mounted with Prolong gold (Invitrogen) and cured for 24 h.

### Confocal and 3D structured illumination microscopy (SIM) imaging

Confocal imaging was performed on the Cured samples by a Zeiss LSM710 using a Plan-Apochrombat 63X/1.40 and an alpha Plan-APO 100X/1.46 oil objective lens (Carl Zeiss). 3D SIM imaging was performed with a Zeiss LSM710 Elyra P1 using an alpha Plan-APO 100X/1.46 oil objective lens. A laser at 561 nm (200 mW) was used for Alexa 568 (Invitrogen). A z-stack was acquired from 42 optical sections with a 200 nm interval. Each section was imaged using five rotations with a 51 nm grating period. 3D SIM Images were rendered using Zen 2012 SP2 software.

### Sperm sample preparation for cryo-electron microscopy

Epididymal spermatozoa from adult male mice (wild type and *Efcab9*^−/−^ in the C57BL/6 background) were collected by swim-out from caudal epididymis^40^. Briefly, male mice were euthanized, and the cauda isolated from the mouse carcass and placed into a 1.5 mL tube with HS medium at RT. To retrieve the mature spermatozoa, the caudal epididymis was cut into several pieces with a scalpel and placed into a 37 °C incubator for 10–30 min to let the sperm swim-out into the HS buffer. Then, the sample was placed at RT for 30 min to let the debris sediment by passive sedimentation, before separating the supernatant with swimming sperm cells from the debris. The supernatant with the sperm was washed one time in PBS, which involved centrifugation at 700 *g* for 5 min at RT.

Human sperm were allowed to settle at the base of a conical tube and the excess buffer was removed. The sperm sample was then passed three times through a Balch ball bearing homogenizer (Isobiotech, 15 μm clearance).

### Cryo-sample preparation for cryo-ET

Small aliquots of freshly prepared mouse sperm at a concentration of 1–5 × 10^6^ cells/mL were gently mixed with ten-fold concentrated, BSA-coated 10 nm colloidal gold solution (Sigma Aldrich) at 3:1 ratio, before applying 4 µL of the solution to a glow-discharged (30 s at 35 mA) copper R 2/2 200-mesh holey carbon grid (Quantifoil Micro Tools). The grids were blotted manually from the back side with Whatman filter paper #1 for 2–4 s, before plunge-freezing the grid in liquid ethane using a homemade plunge-freezer. Grids were stored under liquid nitrogen until either further preparation by cryo-focused ion beam (FIB) milling or imaging by cryo-ET. For mechanical support, grids were mounted into Autogrids (Thermo Fisher).

3 μl of the human sperm sample were applied to glow-discharged copper R2/2 200-mesh holey carbon grid (Quantifoil Micro Tools) and plunge frozen in liquid ethane using an automatic plunge freezer (Vitrobot, FEI, blot force 8, blot time 8 s, Whatman filter paper #1).

### Cryo-electron tomography

Tilt series of whole or cryo-FIB milled mouse sperm flagella were acquired using a Titan Krios (Thermo Fisher Scientific) operated at 300 keV with post-column energy filter (Gatan) in zero-loss mode with 20 eV slit width. Images were recorded using a K3 Summit direct electron detector (Gatan) in counting mode with dose-fractionation (12 frames, 0.05 s exposure time per frame, dose rate of 28 electrons/pixel/s for each tilt image). Tilt series were collected using SerialEM^41^ with the Volta Phase Plate and a target defocus of −0.5 μm. Images were recorded at 26kX magnification resulting in a pixel size of 3.15 Å. Dose-symmetric tilt series^42^ were recorded under low-dose conditions, ranging from ±60° with 2° angular intervals with the total electron dose limited to ~100 e^−^/Å^2^.

Frozen grids of human sperm were loaded into a Jeol3100 TEM operating at 300 kV equipped with an in-column energy filter and a direct electron detector (K2, Gatan). Dose-fractionated, bi-directional tilt series were acquired using SerialEM^41^ with the following parameters: angular increment 1.5°, angular range about ±60° starting at −20°, energy filter slit width 30 eV, nominal magnification 10kX resulting in a detector pixel size of 3.98 Å (which was binned by ×2 resulting in a pixel size of 7.96 Å in the reconstruction), defocus −2.5 μm, exposure time 1 s × 1/cos(tilt angle), fraction interval 0.2 s, dose rate 1 e^−^/Å^2^/s, total dose ~80 e^–^/Å^2^.

### Cryo-FIB milling of mouse sperm

For lamella (section) prepared by cryo-FIB-milling, clipped grids (modified Autogrids with FIB notch) with plunge-frozen mouse sperm were transferred to an Aquilos dual-beam instrument with cryo-sample stage (Thermo Fisher Scientific). Two layers of platinum were added to the sample surface to enhance sample protection and conductivity (sputter-coater: 1 keV and 30 mA for 20 s; gas injection system: when needed, heated up to 28 °C, and then deposited onto the grid for 15 s)^43^. Scanning electron beam imaging was performed at 2 kV and 25 pA, and Gallium ion beam imaging for targeting was performed at 30 kV and 1.5 pA. The target region, i.e., a sperm flagellum, was oriented for milling by tilting the cryo-stage to a shallow angle of 14–16° between the ion beam and the grid. Cryo-FIB milling was performed using a 30 keV gallium ion beam with a current of 30 pA for bulk milling, 30 pA for thinning, and 10 pA for final polishing, resulting in 100–200 nm thick self-supporting lamella, that could then be imaged by cryo-ET.

### Image processing of cryo-ET data

For tilt series of both mouse and human sperm flagella, movie frames were aligned using Motioncor2 1.2.3^44^. The IMOD software^45^ was used to align the tilt serial images using the 10 nm gold particles as fiducial markers and to reconstruct the tomograms by weighted back-projection. For subtomogram averaging, the repeating units were picked manually from raw tomograms. The repeat orientation was determined based on the polarity of the axoneme at the core of the sperm flagella. The alignment and missing-wedge-compensated averaging were performed using the PEET software^46^. After initial averaging a two-fold symmetry was applied. Visualization of the 3D structures of the averaged repeat units was done using the UCSF Chimera software package^47^. Mass estimations from a repeat unit in the subtomogram averages were calculated using the average density of 1.43 g/cm^3^ for proteins^48^ and normalization of the isosurface-rendering threshold in Chimera. The number of tomograms of whole cell and lamella, number of averaged repeats and estimated resolutions of the averages (“Gold standard” FSC at 0.143 and 0.5 criterion), are summarized in Supplementary Table 2.

### Structure fitting and modeling of CatSper complex

The single particle cryo-EM structure and corresponding atomic model of isolated CatSper complexes^18^ were fitted as a rigid body into the subtomogram averaged cryo-ET structure of the in situ CatSper complex using the “fit-in-map” functionality in UCSF chimera^47^. The relative orientations of the CatSper complexes were determined by sequential fitting preceded by rough manual placement. The obtained composite was used to visualize the relative positions of structural models in the zigzag map and to predict potential molecular interactions between the CatSper complexes.

### Analysis of mouse sperm motility and flagellar beating in 4D

For 4D analysis, mouse sperm were washed twice in HS medium and resuspended to a final concentration of 1–2 × 10^6^ cells/mL either under non-capacitating (HS medium) or under capacitating (HS medium, 15 mM NaHCO_3_^−^, 5 mg/mL BSA) conditions. To induce capacitation in vitro, sperm were incubated for 90 min at 37 °C and 5% CO_2_. 4D motility analysis was done at 37 °C and 5% CO_2_ using an off-axis transmission digital holographic microscope DHM^TM^ T-1000 (Lyncée Tec SA, Geneva, Switzerland) equipped with a 666 nm laser diode source, a 20X/0.4 NA objective and a Basler aca1920–155 um CCD camera (Basler AG, Ahrensburg, Germany). Holographic imaging was performed as previously described^33^. In short, mouse sperm were placed in a 100 µm deep chamber slide (Leja) and were recorded at 100 Fps. Offline processing was done using proprietary Koala (Vers. 6; Lyncée Tec SA) and open-source Spyder (Python 3.6.9) software. Using Koala software, *xy*-plane (parallel to the objective slide) projection images of sperm were numerically calculated at different focal planes (*z*-height)^49,50^, followed by sperm head tracking using Spyder to receive *x*, *y* and *z*-coordinates for the entire trajectory. Using these coordinates, motility parameters including 3D curvilinear velocity (VCL, in μm/s) and 2D amplitude of lateral head displacement (ALH, in μm) were analyzed. For each condition, 15 free-swimming single sperm were analyzed using three males from each genotype (wild type, *Efcab9*^*−/−*^, *Catsper1*^*−/−*^).

For 4D flagellar beating analysis, a macro written in Igor Pro^TM^ Vers. 6.36 (Wavemetrics) was used to perform frame-by-frame tracking of flagellar images in stacks of reconstructed *xy*-projections (8-bit TIFF format, 100 Fps, 10 Frame Time) with a resolution of 800 × 800 pixels as well as automatic brightness and contrast adjustments applied by ImageJ V1.50i (National Institutes of Health). A P/U value (3.7466), which is defined as the quotient from the objective magnification (×20) and the pixel size (5.34 μm) of the camera (Basler aca1920–155 μm), was used to convert pixel to micrometer. Calculation of *z*-coordinates was performed utilizing the received *x, y*-coordinates from flagellar traces and Koala. A specific script in Spyder was used to load flagellar *x, y*-coordinates into Koala. Smoothing of *z*-plane data was conducted with Igor Pro^TM^ by fitting to 7th order polynomials. The determination of the distance along the flagellum in the *xy* projections (Dx, y) was carried out geometrically from adjacent pairs of *x*, *y-*coordinates, also using a macro in Igor Pro^TM^.

4D visualization of sperm flagellum and sperm swimming trajectories with respect to the laboratory-fixed frame of reference (*x*, *y*, *z*) was done using OriginPro 2020 software (OriginLab Corporation). Therefore *x*, *y*, and *z*-coordinates of head and flagellar tracking were imported to the software. Analysis was performed for one whole beat-cycle, but for better illustration only, every 6th flagellar excursion between maxima of one beat cycle was illustrated (frame 0, 6, …, 42, every 60 ms) in Fig. 6. The associated movies (Supplementary Movie 5 and 6) of reconstructed trajectories of free-swimming single sperm were created with Cinema4D Vers. 18 (Maxon) using *x*, *y*, and *z*-values of the 4D head tracking. Adobe After Effects software Vers. CS6 (Adobe Systems Software Ireland Limited) was used for video composing and time duration adding. In each supporting video two different perspectives were used to show the 3D movement of sperm during 1 s record. The rolling ball represents the sperm head, and the color code of the trajectory displays the *z*-excursion.

### Quantification and statistical analysis

Statistical analyses were carried out with GraphPad Prism 9 (Statcon GmbH) as indicated in the individual figure legend. Differences were considered significant at *p* < 0.05; exact *p* values were provided in the figure legends. Numerical results are presented as medians and interquartile ranges with *n* = number of determinations and *N* = number of independent experiments.

### Reporting summary

Further information on research design is available in the Nature Research Reporting Summary linked to this article.

## Supplementary information

Supplementary Information

Description of Additional Supplementary Files

Supplementary Movie 1

Supplementary Movie 2

Supplementary Movie 3

Supplementary Movie 4

Supplementary Movie 5

Supplementary Movie 6

Reporting Summary

## Data Availability

The data that support this study are available from the corresponding authors upon reasonable request. The averaged 3D structure of CatSper channel from mouse sperm flagella has been deposited in the Electron Microscopy Data Bank (EMDB) under accession code EMD-24210 (wild type), EMD-26206 (*Efcab9*^−/−^ /) and EMD-26207 (*Efcab9*^−/−^ \). Source data are provided with this paper.

## Source data

Source Data

## Materials availability

DNA constructs generated in this study and *Efcab9*^−/−^ mouse line are available to distribute upon approval of the institutional MTA. There are restrictions to the availability of SLCO6C1 antibodies due to limited quantity.
