# Supplementary material for: 3D structure and in situ arrangements of CatSper channel in the sperm flagellum
Source: Nat Commun. 2022 Jun 17;13:3439. doi: 10.1038/s41467-022-31050-8 (PMC9205950; doi:10.1038/s41467-022-31050-8)
Supplement: Supplementary file 1 — Supplementary Information [file 41467_2022_31050_MOESM1_ESM.pdf]

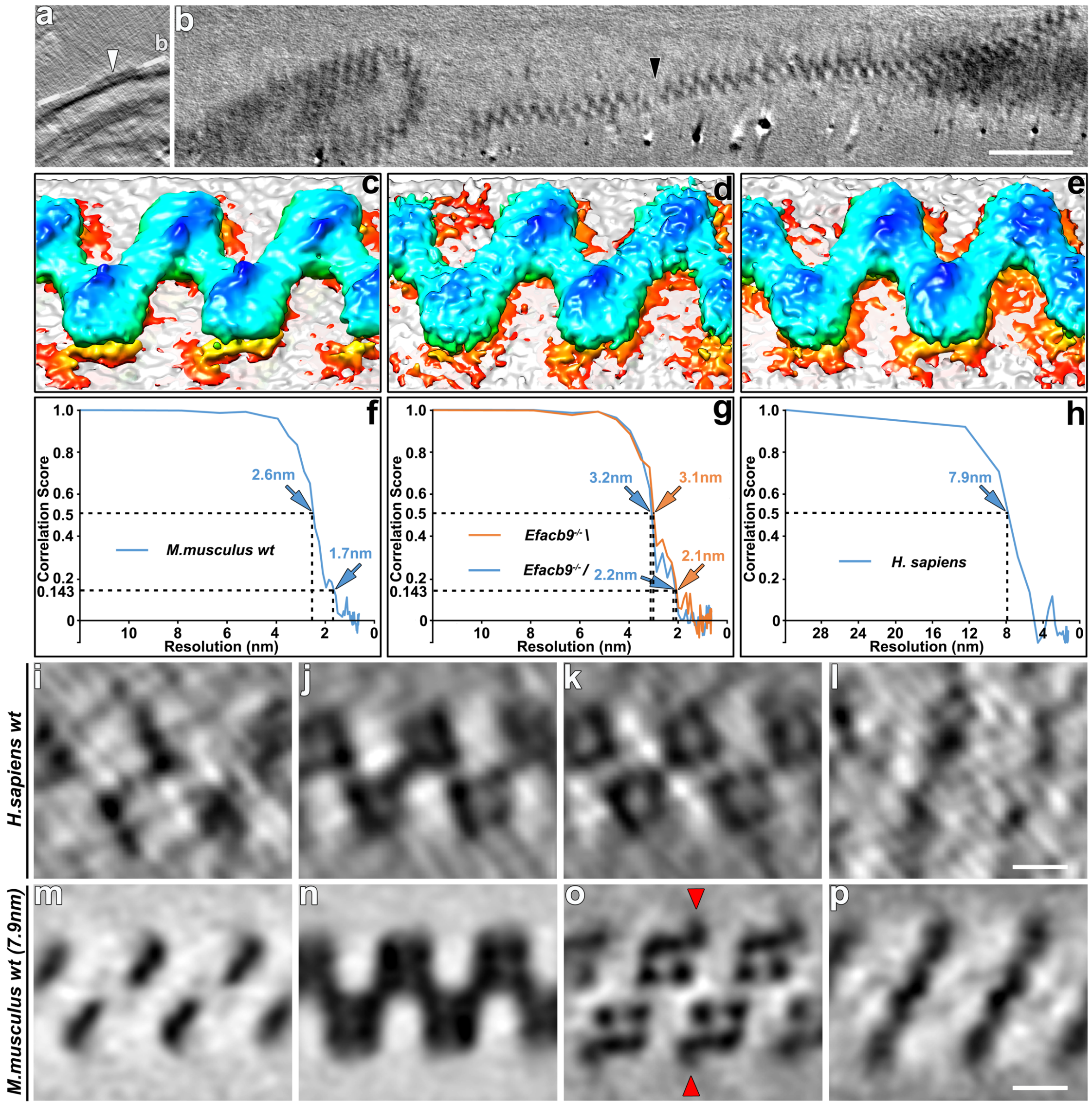

Supplementary Fig. 1 Tomographic reconstruction and subtomogram average of sperm flagella, and resolution measurements. a-b. Tomographic slices showing a cross-sectional (**a**) and top view (**b**) of an *Efcab9*$^{-/-}$ sperm flagellum. The white arrowhead in (**a**) indicates the CatSper structure, and in (**b**) the black arrowhead indicates the interruption of the zigzag row. **c-e.** 3D isosurface renderings of the averaged extracellular CatSper channels in wild type (**c**) and *Efcab9*$^{-/-}$ (/, **d**; \, **e**) mouse sperm. **f-h.** Resolution assessment of the CatSper complex subtomogram averages based on the Fourier shell correlations for mouse wild type (**f**), mouse

Efcab9$^{-/-}$ (**g**), and human (**h**) sperm flagella. **i-o.** Tomographic slices show top views of the averaged CatSper complex in human (**i-l**) and mouse (**m-p**) sperm flagella. Note that the mouse average is shown at 7.9 nm resolution after low-pass filtering for better comparison with the human CatSper structure. The red arrowheads in panel **o** indicate the visibility of the wing structure at this resolution. Slices show the following structural features (roof ridge: **i** and **m**; canopy roof: **j** and **n**; tetrameric channel pore: **k** and **o;** intracellular domain: **l** and **p**. Scale bars: 100 nm in **a, b**; 10 nm in **i-p.**

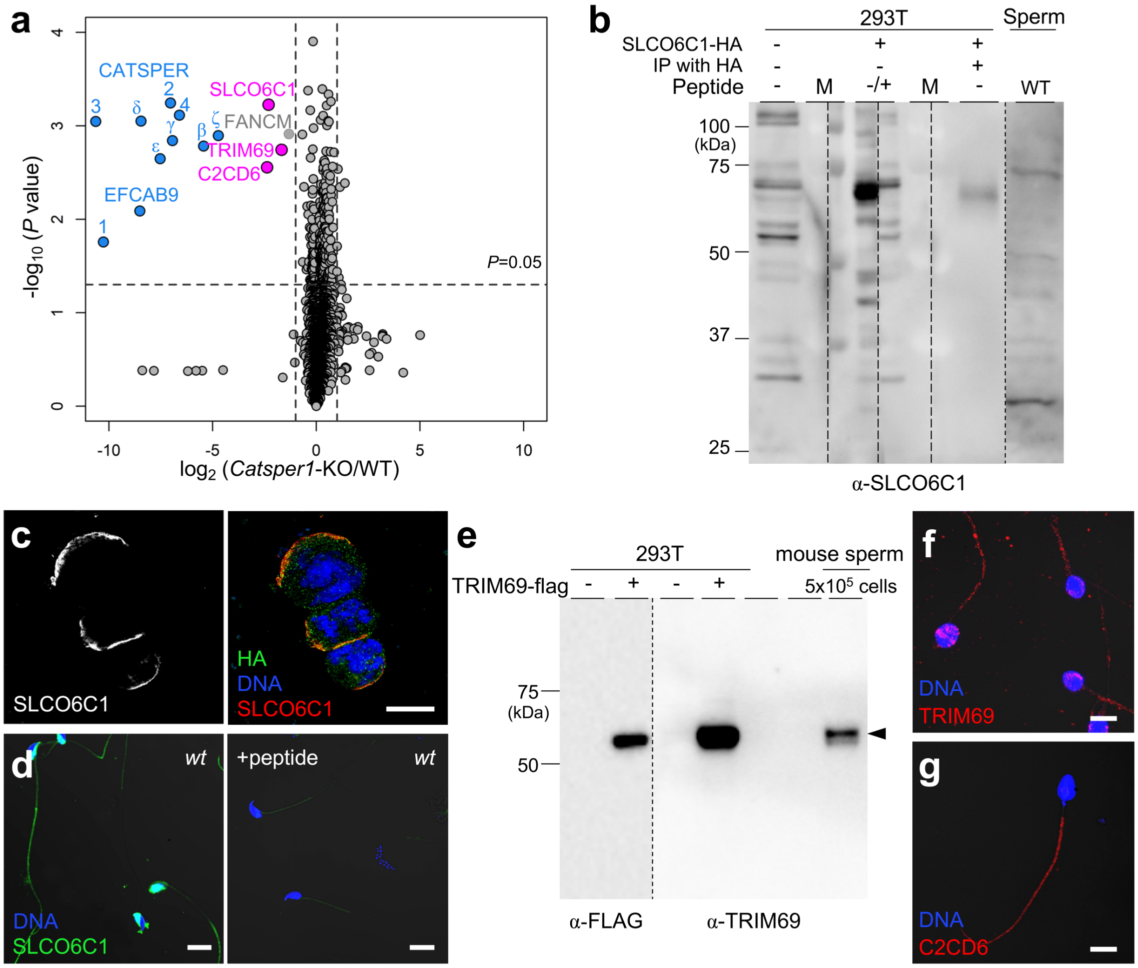

Supplementary Fig. 2 Four candidate CatSper-associated proteins examined, and validation of specificity of antibodies used in this study. a. A volcano plot presentation of the whole cell comparative proteomic analysis between wild type and *Catsper1*$^{-/-}$ mouse sperm[12]. Each protein is represented as a dot and mapped according to its average fold change in *Catsper1*$^{-/-}$ over wild type spermatozoa on the x axis and one-tail t test *p* value on the y axis. In addition to the previously reported CatSper components (blue dots), the protein levels of four additional proteins (pink dots: C2CD6, SLCO6C1, TRIM69, and gray dot: FANCM) were found significantly reduced in *Catsper1*$^{-/-}$ sperm. **b.** Detection of recombinant mouse SLCO6C1 by immunoblotting with SLCO6C1 antibody. **c.** 3D SIM images of immunostained recombinant

SLCO6C1 in 293T cells expressing flag-SLCO6C1-HA. **d.** Confocal images of wild type mouse sperm immunostained by anti-SLCO6C1 antibody before (left) or after preabsorption (right) with epitope peptide. **e.** Detection of recombinant and native mouse TRIM69 proteins by immunoblotting with TRIM69 antibody. **f-g.** Confocal immunofluorescence localization of TRIM69 (**f**) and C2CD6 (**g**) in human sperm. Sperm head counterstained with Hoechst (blue). Scale bars, 100 μm in **b**; 10 μm in **c**; 5 μm in **f**, **g.** All immunoblotting and immunostaining images are representative images from three biological and technical replicates (n=3).

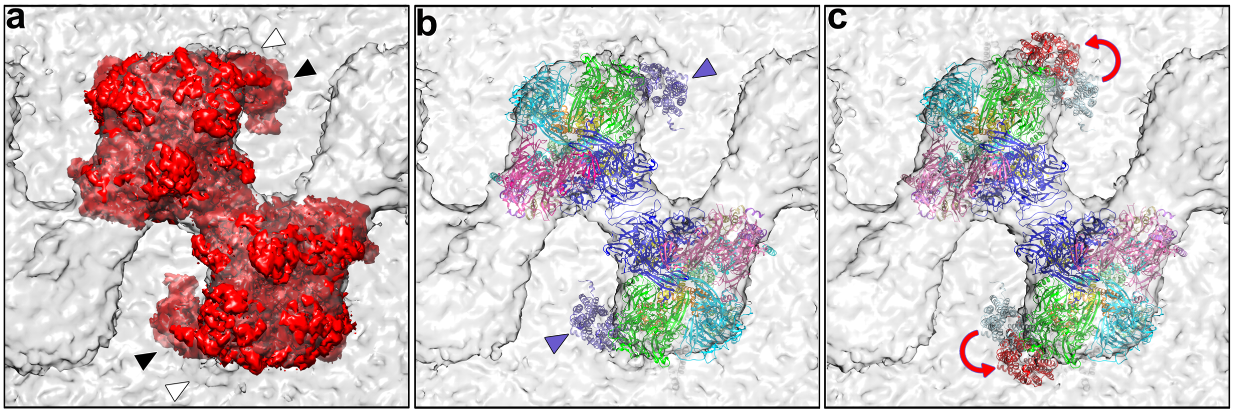

Supplementary Fig. 3 *In situ* position of SLCO6C1 in wild type mouse sperm. a. Top view of two single particle cryo-EM density maps (*red*) of CatSper complex[18] docked into the averaged cryo-ET map of *in situ* CatSper complex (*gray*). Black arrowheads indicate the position of SLCO6C1 observed in the single particle cryo-EM map of isolated CatSper complex, and white arrowheads indicate the *in situ* position of SLCO6C1. **b-c.** Top view of the atomic models in ribbon presentation of two CatSper complexes (see Fig. 5 for color-code of subunits) docked into the averaged *in situ* cryo-ET map. Purple arrowheads in (**b**) indicate the position of SLCO6C1 observed by single particle cryo-EM. Arrows in (**c**) indicate the correction of the SLCO6C1 position based on our cryo-ET map by rigid body fitting.

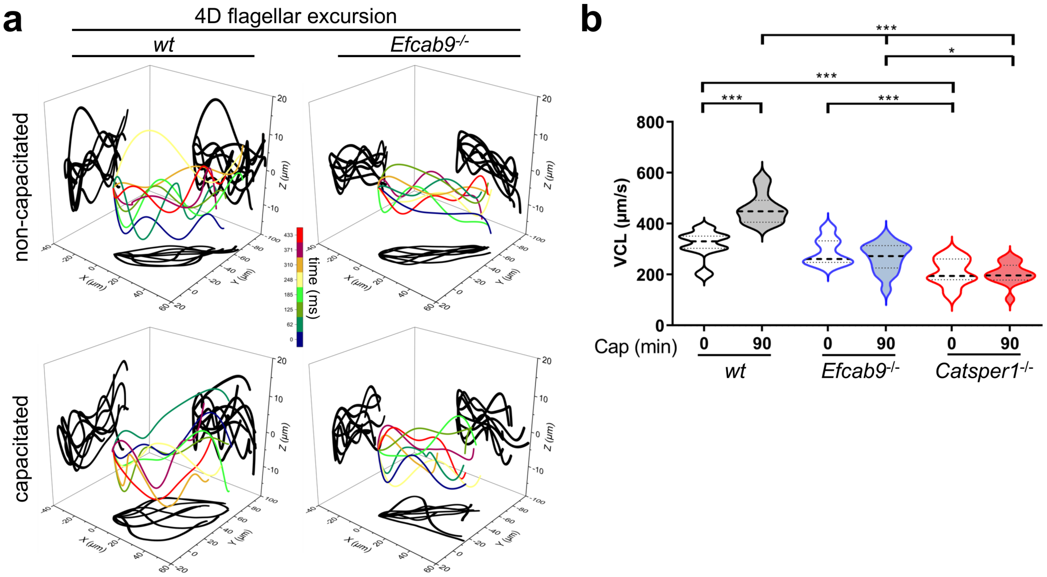

Supplementary Fig. 4 Capacitation-induced CatSper activation leads to an increase in *xy*-excursion and curvilinear velocity. a. 4D flagellar beating waveforms from wild type and *Efcab9*$^{-/-}$ sperm generated using DHM imaging. The head position is set at 0 to better visualize the flagellar waveform and the flagellar trace is projected into the *xy*-, *xz*- and *yz*-planes. **b.** Curvilinear velocity (VCL, µm/s) measured from DHM imaging of free-swimming sperm from wild type, *Efcab9*$^{-/-}$ and *Catsper1*$^{-/-}$ mice before (0 min) and after capacitation (90 min). n=15 each group of three animals, a two-way repeated-measures ANOVA statistical test was performed, *p (0.0252) < 0.05, ***$p < 0.001$, p value below 0.001 cannot be given as exact value; the medians (thick dash lines) and interquartile ranges (thin dash lines) are shown.

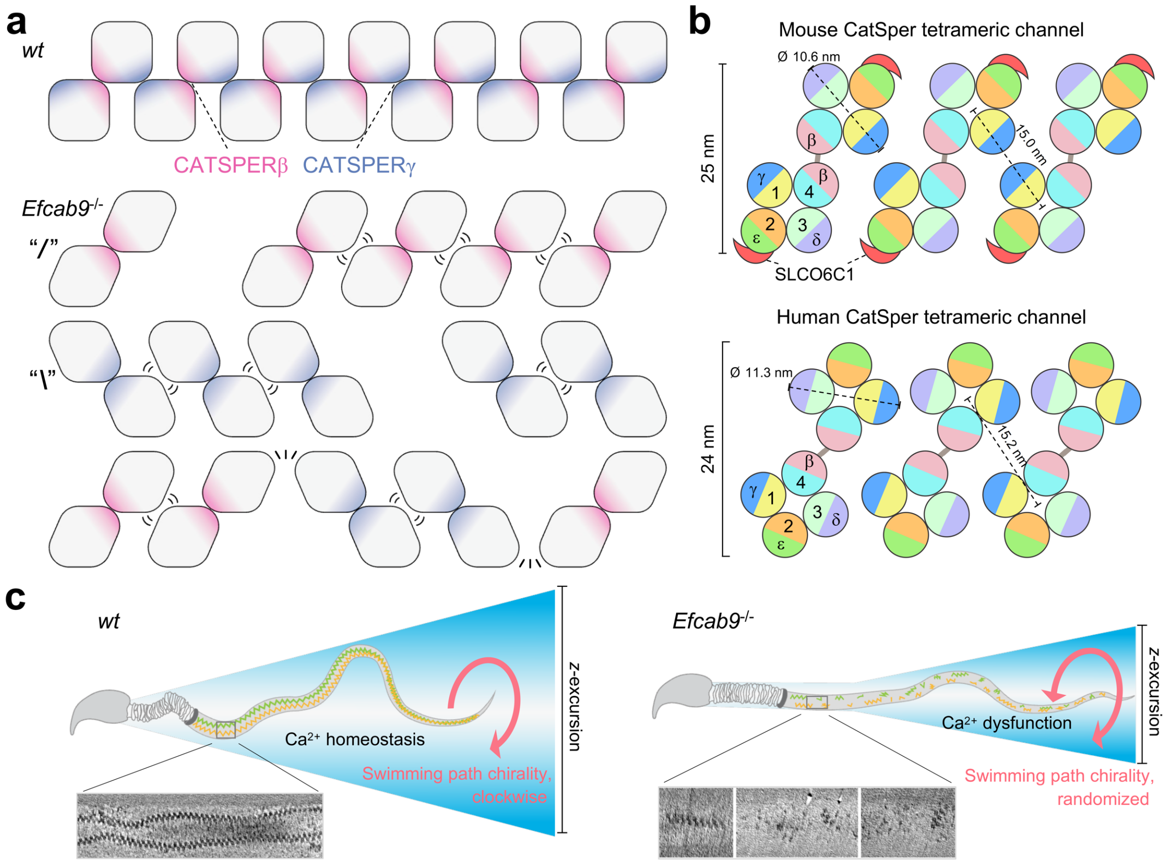

Supplementary Fig. 5 Orientation of CatSper channels and high-order arrangements within the zigzag row and models of their relationship with sperm motility.

a. Cartoons showing the zigzag arrangement of CatSper channel in wild type and *Efcab9*$^{-/-}$; in the absence of the EFCAB9-CATSPERζ subcomplex, the mutant CatSper complex shows two "distorted" states: (/) with weakened inter-dimer interactions between two CATSPERγ subunits (*blue*) of neighboring complexes, and (\) with weakened dimeric interaction between CATSPERβ (*pink*) of neighboring complexes. Interactions between (/) and (\) clusters seem to be unfavorable (bottom row) and result in further fragmentation of the zigzag row. **b.** Schematic models depicting the similarities and differences of the CatSper tetrameric channels in mouse and human sperm flagella. Mouse CatSper tetrameric channel has an outwardly directed wing (SLCO6C1) associated with CATSPER2/ε[18]. Two staggered neighboring channels along the

zigzag string are connected between CATSPERβ of each channel complex that are 180º rotated to each other. The angle between CATSPER4/β within a dimer along the zigzag string is different between mouse and human sperm. **c.** Working model illustrating the higher-order arrangement of the CatSper channel complexes along the flagellum and their relation to sperm motility and swimming path chirality. In wild type sperm, the CatSper channels are arranged in continuous zigzag-rows that are aligned with the axis of the flagellum. In *Efcab9*$^{-/-}$ sperm, the rows are fragmented into short clusters that are often misaligned relative to the flagellar axis.

Supplementary Table 1. The estimated molecular weight of mouse and human CatSper channel components and their domains.

		Mouse[1]							Human[2]						
	Name	M.W. (kD)	Extracellular Domain (kD)			Cytoplasmic Domain (kD)			M.W. (kD)	Extracellular Domain (kD)			Cytoplasmic Domain (kD)		
Pore subunits	CATSPER1	79	6	24	472	52	132	356	90	6	22	419	67	147	365
	CATSPER2	69	7			38			62	6			38		
	CATSPER3	46	6			15			46	5			10		
	CATSPER4	51	5			27			54	5			32		
TM auxiliary subunits	CATSPERβ	126	120	431		2	19		127	96	397		24	43	
	CATSPERγ	132	122			8			133	119			8		
	CATSPERδ	91	81			7			91	80			7		
	CATSPERε	114	108			2			110	102			4		
Non-TM auxiliary subunits	CATSPERζ	23	N.A.	N.A.		23	49		23	N.A.	N.A.	N.A.	23	47	
	EFCAB9	26	N.A.			26			24	N.A.			24		
New associated proteins	C2CD6	63	N.A.	17		63	156		71	N.A.	N.A.		71	128	
	TRIM69	57	N.A.			57			57	N.A.			57		
	SLCO6C1	79	17			36									N.A.

[1]Molecular weight estimation of mouse CatSper is based on the Universal Protein Resource (UniProt) and Lin et al.[18]. [2] Estimation of human CatSper is based on UniProt only.

Supplementary Table 2. Data processing summary for electron tomograms and sub-tomogram averaging.

Parameters (for strain)	*Mus musculus* (mouse)				*Homo sapiens* (human)
	wild type		*Efcab9*$^{-/-}$		
Sample type	whole cell	cryo-FIB lamella	whole cell	cryo-FIB lamella	whole cell
TEM/Camera	Krios/K3/VPP		Krios/K3/VPP		Jeol/K2/Defocus
Pixel size [Å]	3.15		3.15		7.96 (binned)
# of collected tomograms	57	22	20	83	1
# of tomograms with visible CatSper complex	4	7	4	30	1
# of tomograms used for sub-tomogram average	4	7	1	18	1
Symmetry	C2		C2		C2
Averaged repeats	2542		536 '/' and 1072 '\'		32
Estimated resolution [nm] (FSC criterions in bracket)	1.7 (0.143) and 2.6 (0.5)		2.2 '/'; 2.1'\' (0.143) and 3.2 '/'; 3.1 '\' (0.5)		7.9 (0.5)

EMDB accession: EMD-24210 [https://www.ebi.ac.uk/pdbe/entry/emdb/EMD-24210] (wild type); EMD-26206 [https://www.ebi.ac.uk/pdbe/entry/emdb/EMD-26206] (Efcab9$^{-/-}$ /) and EMD-26207 [https://www.ebi.ac.uk/pdbe/entry/emdb/EMD-26207] (Efcab9$^{-/-}$ \).
