# Supplementary material for: 3D structure and in situ arrangements of CatSper channel in the sperm flagellum
Source: Nat Commun. 2022 Jun 17;13:3439. doi: 10.1038/s41467-022-31050-8 (PMC9205950; doi:10.1038/s41467-022-31050-8)
Supplement: Supplementary file 2 — Description of Additional Supplementary Files [file 41467_2022_31050_MOESM2_ESM.pdf]

Description of Additional Supplementary Files

File Name: Supplementary Movie 1
Description: **In-cell cryoET reconstruction shows the higherorder arrangement of CatSper channel complexes in a wild type mouse sperm flagellum.** Animated tomographic slices through a representative tomogram (also shown in Fig. 2c and d) first highlight the ECDs of two CatSper rows (arrowheads) in cross view of the flagellum, then rotate into a side view (with the proximal side of the flagellum on the left), and further into the top view. Then sequential tomographic slices are shown first moving away from the membrane further into the extracellular space, before reversing the direction, crossing through the membrane into the cytoplasm and back.

File Name: Supplementary Movie 2
Description: ***In situ* cryoET reconstruction shows the disruption of the higher-order arrangement of CatSper channel complexes in an Efcab9$^{-/-}$ mouse sperm flagellum.** Animated tomographic slices through a representative tomogram (also shown in Fig. 2q and r) first highlight the ECDs of a CatSper row (arrowheads) in cross view of the flagellum, then rotate into a side view (with the proximal side of the flagellum on the left), and further into the top view. Then sequential tomographic slices are shown first moving away from the membrane further into the extracellular space, before reversing the direction, crossing through the membrane into the cytoplasm and back. Backslash (\) and forward slash (/) are pseudo-colored in blue and pink, respectively. Black arrowheads indicate the interruptions of the CatSper zigzag row

File Name: Supplementary Movie 3
Description: **Subtomogram averaging reveals the 3D structure and arrangement of the CatSper complexes in wild type mouse sperm.** Sequential tomographic slices from the intracellular region (I), through the membrane to the extracellular region showing the tetrameric channel (C) with attached wing structure (arrowheads), canopy roof (R) and roof ridge (Rr), before revealing the 3D isosurface rendering representation that rotates from the extracellular to the intracellular view and back, ending in the side view.

File Name: Supplementary Movie 4
Description: **A pseudoatomic model of the *in situ* CatSper complex was generated by docking of the single particle structure of isolated monomeric CatSper to the subtomogram averaged *in situ* mouse CatSper complexes, revealing the 3D structure of the CatSper complex dimer, higher-order arrangement and molecular interactions between complexes.** Sequential tomographic slices from the intracellular region (I), through the membrane (M) to the extracellular region (E) as indicated by the white line in the inset (also shown in Fig. 3a), followed by docking of the atomic models of three mouse CatSper complex structure into the averaged cryo-ET map. Then predicted interactions at the intra- and interdimeric interfaces between CatSper complexes are shown and annotated, together with a top guide view (upper right).

File Name: Supplementary Movie 5
Description: **4D sperm swimming trajectory of noncapacitated sperm.** The swimming trajectories are acquired by tracing sperm head position of wild type, Efcab9$^{-/-}$ and Catsper1$^{-/-}$ sperm (0 min).

File Name: Supplementary Movie 6
Description: **4D sperm swimming trajectory of capacitated sperm.** The swimming trajectories are acquired by tracing sperm head position of wild type, *Efcab9*$^{-/-}$ and *Catsper1*$^{-/-}$ sperm incubated under capacitating conditions (90 min).
