# Supplementary material for: 3D structure and in situ arrangements of CatSper channel in the sperm flagellum
Source: Nat Commun. 2022 Jun 17;13:3439. doi: 10.1038/s41467-022-31050-8 (PMC9205950; doi:10.1038/s41467-022-31050-8)
Supplement: Supplementary file 9 — Reporting Summary [file 41467_2022_31050_MOESM9_ESM.pdf]

nature portfolio

Corresponding author(s): Jean-Ju Chung, Daniela Nicastro

Last updated by author(s): May 20, 2022

Reporting Summary

Nature Portfolio wishes to improve the reproducibility of the work that we publish. This form provides structure for consistency and transparency in reporting. For further information on Nature Portfolio policies, see our Editorial Policies and the Editorial Policy Checklist.

Statistics

For all statistical analyses, confirm that the following items are present in the figure legend, table legend, main text, or Methods section.

n/a	Confirmed	
☐	☒	The exact sample size (*n*) for each experimental group/condition, given as a discrete number and unit of measurement
☐	☒	A statement on whether measurements were taken from distinct samples or whether the same sample was measured repeatedly
☐	☒	The statistical test(s) used AND whether they are one- or two-sided<br>*Only common tests should be described solely by name; describe more complex techniques in the Methods section.*
☒	☐	A description of all covariates tested
☒	☐	A description of any assumptions or corrections, such as tests of normality and adjustment for multiple comparisons
☒	☐	A full description of the statistical parameters including central tendency (e.g. means) or other basic estimates (e.g. regression coefficient) AND variation (e.g. standard deviation) or associated estimates of uncertainty (e.g. confidence intervals)
☐	☒	For null hypothesis testing, the test statistic (e.g. *F*, *t*, *r*) with confidence intervals, effect sizes, degrees of freedom and *P* value noted<br>*Give P values as exact values whenever suitable.*
☒	☐	For Bayesian analysis, information on the choice of priors and Markov chain Monte Carlo settings
☒	☐	For hierarchical and complex designs, identification of the appropriate level for tests and full reporting of outcomes
☒	☐	Estimates of effect sizes (e.g. Cohen's *d*, Pearson's *r*), indicating how they were calculated

Our web collection on statistics for biologists contains articles on many of the points above.

Software and code

Policy information about availability of computer code

Data collection	SerialEM (3.8.2) was used to collect cryo-ET data. Zeiss Zen software (2012 SP2) was used to collect immunostainning data. Koala (Vers. 6) was used for 4D flagellar waveform and swimming pattern data acquisition.
Data analysis	Motioncor2 (1.2.3), IMOD (4.9.3), PEET (1.10.0); Chimera (1.10.2) were used to analysis and show cryo-ET data.<br>GraphPad Prism 9 Koala (Vers. 6), open-source Spyder (Python 3.6.9), Igor Pro (6.36), OriginPro (2020) and ImageJ (V1.50i) were used to analyzed 4D flagellar waveform and swimming pattern. Adobe After Effects software (Vers. CS6) was used for video composing and time duration adding.

For manuscripts utilizing custom algorithms or software that are central to the research but not yet described in published literature, software must be made available to editors and reviewers. We strongly encourage code deposition in a community repository (e.g. GitHub). See the Nature Portfolio guidelines for submitting code & software for further information.

Data

Policy information about availability of data

All manuscripts must include a data availability statement. This statement should provide the following information, where applicable:

- Accession codes, unique identifiers, or web links for publicly available datasets
- A description of any restrictions on data availability
- For clinical datasets or third party data, please ensure that the statement adheres to our policy

The averaged 3D structure of CatSper channel from mouse sperm flagella has been deposited in the Electron Microscopy Data Bank (EMDB) under accession code EMD-24210 (wild type), EMD-26206 (Efcab9-/- /) and EMD-26207 (Efcab9-/- \).

Field-specific reporting

Please select the one below that is the best fit for your research. If you are not sure, read the appropriate sections before making your selection.

- [x] Life sciences
- [ ] Behavioural & social sciences
- [ ] Ecological, evolutionary & environmental sciences

For a reference copy of the document with all sections, see nature.com/documents/nr-reporting-summary-flat.pdf

Life sciences study design

All studies must disclose on these points even when the disclosure is negative.

Sample size	For cryo-ET, 183 tomograms (182 tomograms from mouse sperm cells, 1 tomograms from human sperm cells) were collected in this study. 31 tomograms (30 tomograms for mouse, 1 for human) were chosen for sub-tomogram average because of the practical limitations of the methodology used and the distribution of structure studied. For immunoblotting and immunocytochemistry analysis, n=3 was chosen as the minimal biological and technical replicate number. For 4D-flagellar waveform analysis and swimming pattern, n=15 per group of 3 animals was chosen as the replicate number. Several million sperm cells per group were used for making grids, coverslips, lysate and imaging. We determined this to be sufficient owing to internal control and low observed variability.
Data exclusions	For cryo-ET, some tomograms were excluded for sub-tomogram average (see in Sample size) because of the practical limitations of the methodology used and the distribution of structure studied. For other data, there is no exclusion from analysis.
Replication	For cryo-ET, immunocytochemistry, 4D-flagellar waveform and swimming pattern analysis, data was collected on different individual sperm samples from at least 3 mice. For immunoblotting, samples are made from at least 3 mice and tested individually. Each data point can therefore be considered as a biological replicate. All replication attempts were successful through the study.
Randomization	Sperm individuals were randomly chosen among each genotype population. For cryo-ET, immunocytochemistry, 4D-flagellar waveform and swimming pattern analysis, data collection were selected at random without bias toward sample location on girds or coverslips, size or fields under the microscope when applicable. For immunoblotting, millions of sperm was randomly collected from the suspension then was lysed for gel running.
Blinding	The persons performing sample preparation and EM imaging were unaware of the sample identity. A subset of the data sets has been analyzed in a double-blind approach, resulting in the same distributions as in the non-blinded analysis. All localization data have been documented and are available upon reasonable request.

Reporting for specific materials, systems and methods

We require information from authors about some types of materials, experimental systems and methods used in many studies. Here, indicate whether each material, system or method listed is relevant to your study. If you are not sure if a list item applies to your research, read the appropriate section before selecting a response.

Materials & experimental systems

n/a	Involved in the study	
[ ]	[x]	Antibodies
[ ]	[x]	Eukaryotic cell lines
[x]	[ ]	Palaeontology and archaeology
[ ]	[x]	Animals and other organisms
[ ]	[x]	Human research participants
[x]	[ ]	Clinical data
[x]	[ ]	Dual use research of concern

Methods

n/a	Involved in the study	
[x]	[ ]	ChIP-seq
[x]	[ ]	Flow cytometry
[x]	[ ]	MRI-based neuroimaging

Antibodies

Antibodies used	Anti-CATSPER1, EFCAB9, C2CD6, SLCO6C1 antibodies are homemade either in Chung lab or previously in Clapham lab. Other antibodies are from commercial sources: TRIM69 (Origene, AP31866PU-N), acetylated tubulin (Sigma, T7451, 0000128058), HA (Cell Signaling Technology, clone 6E2, 2367S), anti-rabbit IgG-HRP (Jackson ImmunoResearch, Code: 115-035-144), anti-goat IgG-HRP (Jackson ImmunoResearch, Code: 705-035-003), and anti-mouse IgG-HRP (Jackson ImmunoResearch, Code: 115-035-146)
Validation	The specificities of homemade CATSPER1, EFCAB9, C2CD6 antibodies were knockout validated (the absence of the signal in knockout animals). from our previous work. Slco6c1 antibody and TRIM69 was validated by peptide absorption in native sample and co-detection of the tagged recombinant protein by the antibody or anti-tag in immunoblotting and inmmunocytochemistry from this manuscript (Suppl fig. 2). Other commercial antibodies (acetylated tubulin and HA) were validated in the manufacturer's website.

Eukaryotic cell lines

Policy information about cell lines

Cell line source(s)	HEK293T cells (human, ATCC CRL-1573)
Authentication	None of the cell lines have been authenticated.
Mycoplasma contamination	Cell lines were not tested for mycoplasma contamination but no indication of contamination was observed.
Commonly misidentified lines (See ICLAC register)	No commonly misidentified cell lines were used.

Animals and other organisms

Policy information about studies involving animals; ARRIVE guidelines recommended for reporting animal research

Laboratory animals	Mice were housed at a temperature of 20–25 C under a 12/12-h light/dark schedule with 2–5 mice per cage. 8-12 weeks old C57/BL6 male mice from wild type, Catsper1-/-, Catsperd-/-, Catsperz-/- and Efcab9-/- were used for sperm collection.
Wild animals	No wild animals were used in this study.
Field-collected samples	No field-collected samples were used in this study
Ethics oversight	Mice were cared according to the guidelines approved by the Institutional Animal Care and Use Committee (IACUC) of Yale University (#20079)

Note that full information on the approval of the study protocol must also be provided in the manuscript.

Human research participants

Policy information about studies involving human research participants

Population characteristics	25-39 years old men were recruited and consented to participate this study. Freshly ejaculated semen samples were obtained by masturbation. All processed samples were normozoospermic with a cell count of at least 30 million sperm cells per mL.
Recruitment	A total of 3 healthy volunteers were recruited randomly. All volunteers showed normal semen analysis at the time of sample collection. There is very small possibility that all volunteers harbor CatSper channel related mutation then affect the channel configuration or conformation.
Ethics oversight	The experimental procedures utilizing human-derived samples were approved by the Committee on Human Research at the University of California, Berkeley, IRB protocol number 2013-06-5395.

Note that full information on the approval of the study protocol must also be provided in the manuscript.
