# Supplementary figures and images for: 3D structure and in situ arrangements of CatSper channel in the sperm flagellum
Source: Nat Commun. 2022 Jun 17;13:3439. doi: 10.1038/s41467-022-31050-8 (PMC9205950; doi:10.1038/s41467-022-31050-8)

## Full scan blot images in Fig. 4c

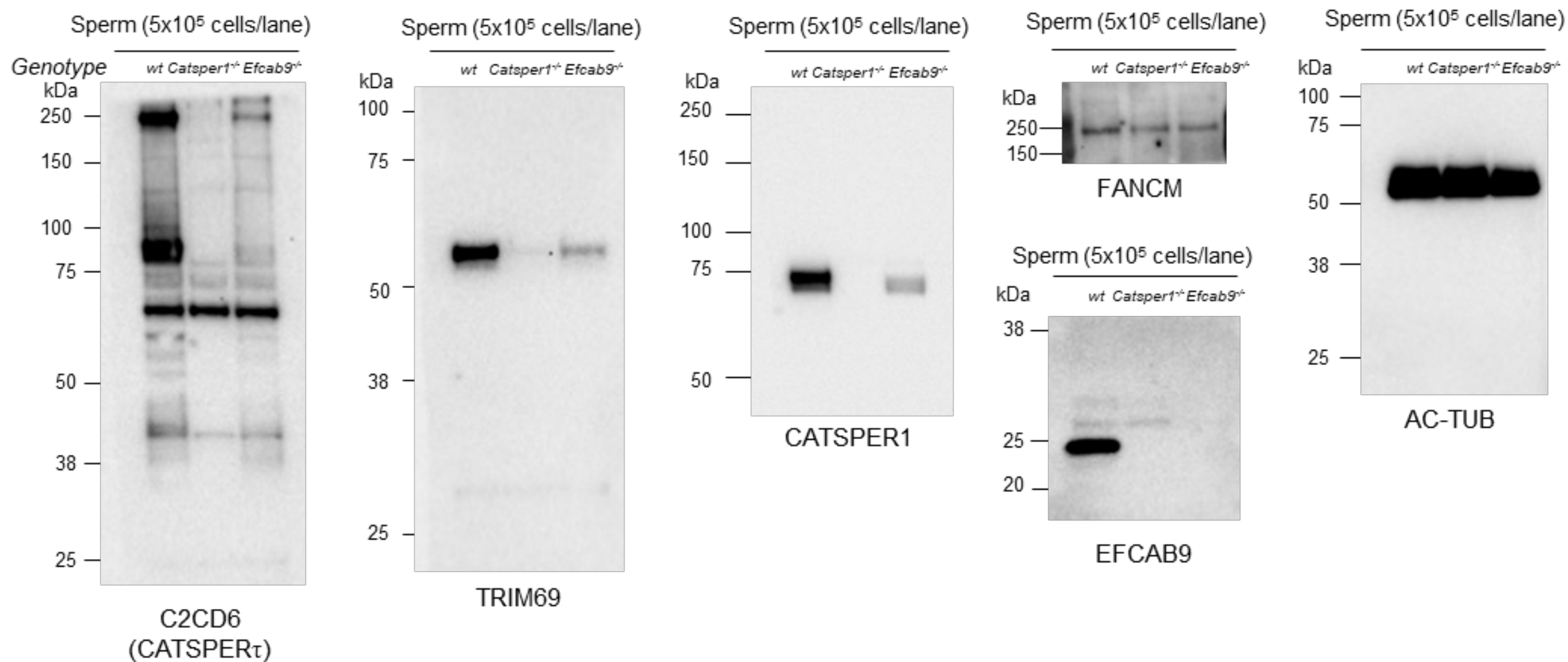

## Full scan blot images in Fig. 4f

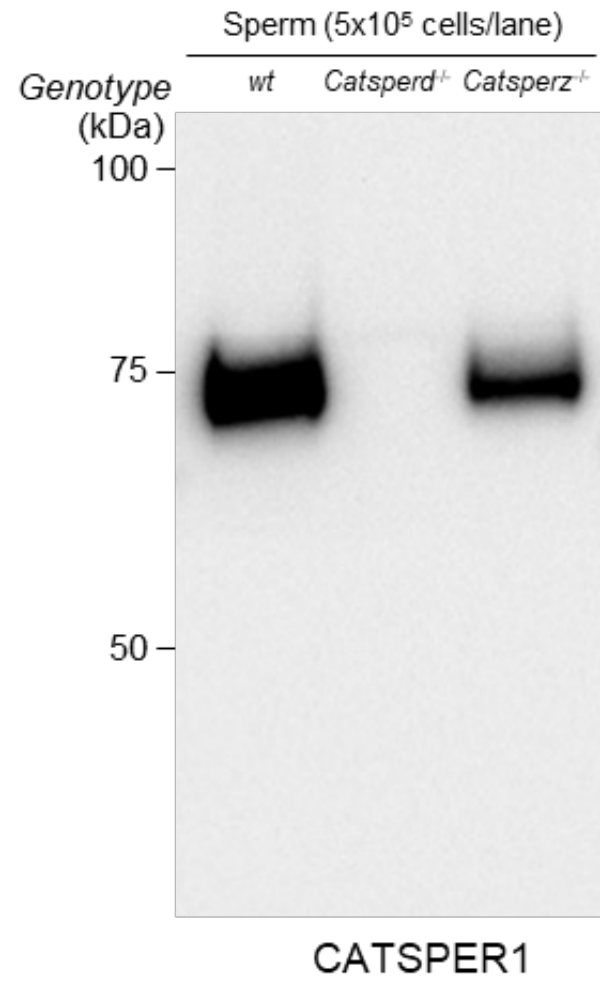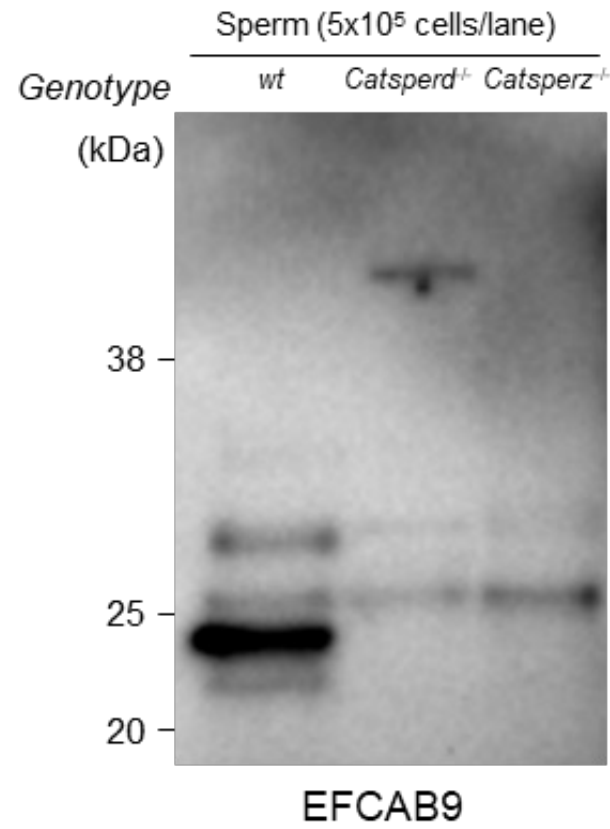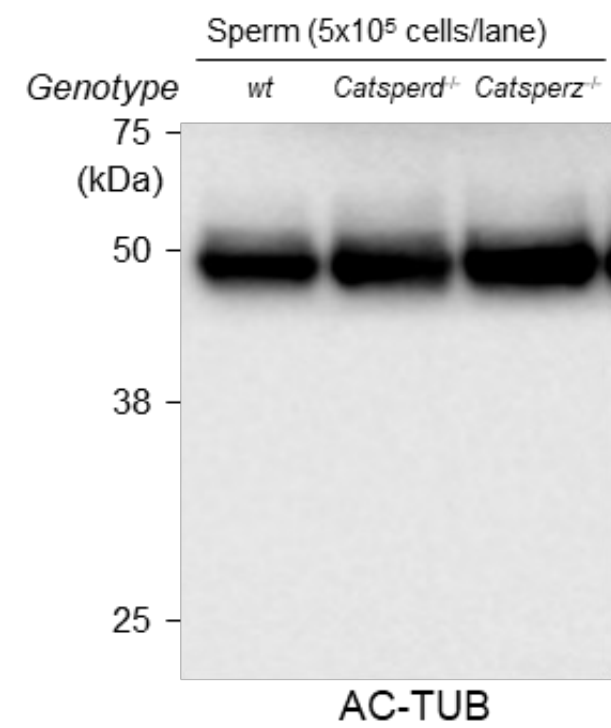

Supplement: Supplementary file 10 — Source Data [file 41467_2022_31050_MOESM10_ESM.pdf]
